# Integrated pest management strategies for cabbage stem flea beetle (*Psylliodes chrysocephala*) in oilseed rape

**DOI:** 10.1111/gcbb.12918

**Published:** 2022-01-16

**Authors:** Patricia A. Ortega‐Ramos, Duncan J. Coston, Gaëtan Seimandi‐Corda, Alice L. Mauchline, Samantha M. Cook

**Affiliations:** ^1^ Biointeractions & Crop Protection Department Rothamsted Research Harpenden Hertfordshire UK; ^2^ School of Agriculture, Policy and Development University of Reading Reading UK

**Keywords:** biocontrol, *Brassica napus*, control threshold, insect pest control, pesticides, sustainable agriculture

## Abstract

Oilseed rape (OSR) is the second largest source of vegetable oil globally and the most important biofuel feedstock in the European Union (EU) but the production of this important crop is threatened by a small insect, *Psylliodes chrysocephala* – the cabbage stem flea beetle (CSFB). The EU ban on use of neonicotinoid seed treatments and resistance of CSFB to pyrethroid insecticides have left farmers with limited control options resulting in drastic reductions in production. Integrated pest management (IPM) may offer a solution. We review the lifecycle of CSFB and the current options available, or in the research pipeline, for the eight IPM principles of the EU Sustainable Use of Pesticides Directive (Directive‐2009/128/EC). A full IPM strategy for CSFB barely exists. Although there are a range of preventative measures, these require scientific validation; critically, resistant/tolerant OSR cultivars are not yet available. Existing monitoring methods are time‐consuming and there are no commercial models to enable decision support based on predictions of migration timing or population size. Available thresholds are not based on physiological tolerances of the plant making it hard to adapt them to changing market prices for the crop and costs of control. Non‐synthetic alternatives tested and registered for use against CSFB are lacking, making resistance management impossible. CSFB control is therefore dependent upon conservation biocontrol. Natural enemies of CSFB are present, but quantification of their effects is needed and habitat management strategies to exploit their potential. Although some EU countries have local initiatives to reduce insecticide use and encourage use of ‘greener’ alternatives, there is no formal process for ranking these and little information available to help farmers make choices. We summarize the main knowledge gaps and future research needed to improve measures for CSFB control and to facilitate development of a full IPM strategy for this pest and sustainable oilseeds production.

## Graphical abstract

Oilseed rape is the second largest source of vegetable oil globally and the primary biofuel feedstock in the European Union, but production is threatened by *Psylliodes chrysocepaha* – the cabbage stem flea beetle (CSFB). Due to the ban on neonicotinoid seed treatments and development of pyrethroid resistance, CSFB can no longer reliably be controlled using insecticides alone. We review the lifecycle of CSFB and the current options available for Integrated Pest Management (IPM). A full IPM strategy for CSFB barely exists. We summarise the main knowledge gaps and future research needed to facilitate development of an IPM strategy for this pest.

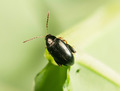

## 1 INTRODUCTION

Oilseed rape (*Brassica napus*, L., OSR) is the second largest source of vegetable oil in the world, after soybean (European Commission, 2018); grown throughout most of the European continent (Table S1), it is the dominant biodiesel feedstock in the European Union (EU), accounting for 39% of total biodiesel feedstock production (USDA, 2019). Its importance as biofuel feedstock has contributed substantially to the rising value of the crop; the additional demand from the energy sector (Directive 2003/30/EC, 2003) expanded the use of the crop, and consequent increase in production from 11.1 Mt in 2003 to 21.4 Mt in 2009 (Faostats, 2021). This, however, led to almost unlimited availability of resource for insect pests. OSR is attacked by a suite of insect pests (Williams, 2010a), which can significantly impact yield (Zheng et al., 2020) and the cabbage stem flea beetle (CSFB, *Psylliodes chrysocephala* L. Chrysomelidae) has been ranked as the most significant biotic threat to OSR cultivation in Europe (Zheng et al., 2020). Due to severe infestations by CSFB, farmers, especially in the UK and northern Europe, are struggling to grow OSR and are opting to grow alternative crops (Andert et al., 2021; Defra, 2017; Wynn et al., 2017). Major reductions in OSR area have led to a decline in production in Europe (Andert et al., 2021; Zheng et al., 2020); the area of OSR harvested in the EU for 2019–2020 was the lowest since 2006–2007 (USDA, 2020). This was mainly attributed to higher pest pressure and decreasing availability of registered active ingredients for chemical control, especially since the ban on neonicotinoid seed treatments (Andert et al., 2021; Zheng et al., 2020).

### 1.1 Ecology of CSFB

**FIGURE 1:**
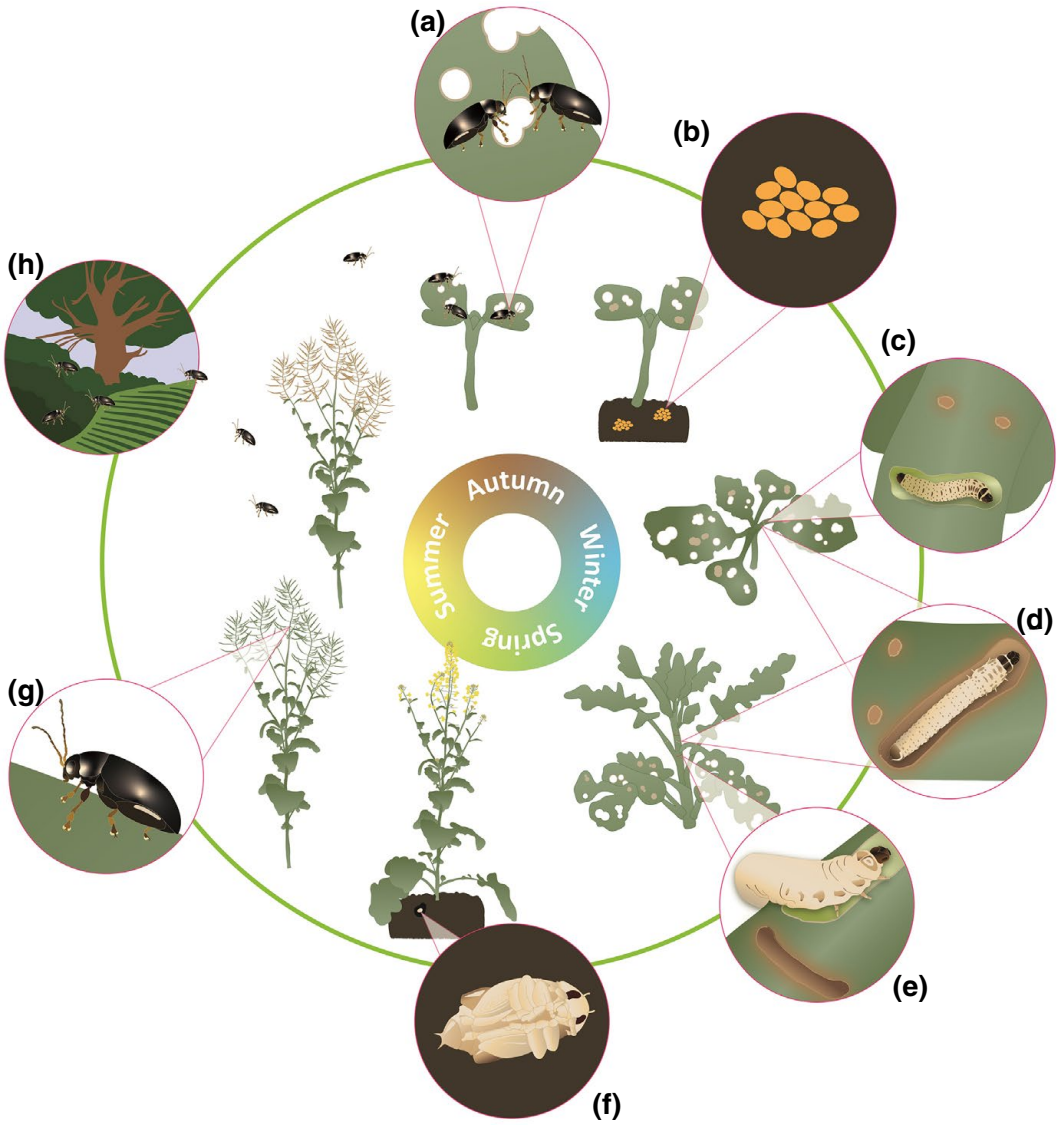
Lifecycle of cabbage stem flea beetle (*Psylliodes chrysocephala*) and damage symptoms caused to oilseed rape (OSR) host plants. (a) adult migration to OSR crops and feeding on cotyledons causing ‘shot‐holing’ symptoms; (b) eggs laid in the soil; (c) first instar larvae mining OSR petioles and petiole scars; (d) second instar larvae mining OSR petioles and petiole scars; (e) third and last instar larvae mining main stem and leaf scar; (f) pupa buried in the soil; (g) new generation adult feeding on OSR stems and pods; (h) adult aestivation in sheltered areas such as hedgerows and woodlands

Adult CSFB are present throughout most of the European continent (CABI, ; Table S1). They are oval in shape, 3.2–4.6 mm long (Bonnemaison & Jourdheuil, 1954), and have 10 antennal segments and thickened hind femurs to enable them to jump to avoid predators (Furth, 1988; Ruan et al., 2020) (see graphical abstract). They are usually black with a blue‐green metallic sheen although a brown variant also occurs (Bonnemaison & Jourdheuil, 1954). Adult beetles migrate to newly sown OSR crops in autumn; they are able to fly up to 3–4 km (Bonnemaison, 1965). Migration flights generally end in October and once in the crop their flight muscles atrophy (Bonnemaison, 1965; Ebbe‐Nyman, 1952). The beetles feed on the cotyledons and young leaves of plants (Figure 1a); after a period of c. 2 weeks, they start to mate and oviposition begins (Alford et al., 2003; Bonnemaison & Jourdheuil, 1954; Sáringer, 1984). Oviposition usually peaks in autumn, when temperatures are between 2 and 16°C, but continues until early spring in mild conditions (Bonnemaison, 1965; Bonnemaison & Jourdheuil, 1954; Mathiasen, Sørensen, et al., 2015; Meuche, 1940; Sáringer, 1984). Eggs are oval, orange, 0.6 mm long and 0.4 mm wide (Bonnemaison & Jourdheuil, 1954; Figure 1b) and are laid in batches in the soil near the host plant (Sáringer, 1984; Vig, 2003). Eggs hatch from September onwards (Alford, 1979; Johnen et al., 2010) but larvae are sensitive to cold winter frosts which could limit their distribution in the furthest north areas of Europe (Mathiasen, Bligaard et al., 2015; Mathiasen, Sørensen, et al., 2015). The neonate larvae tunnel into the plant and feed and develop gregariously in the plant petioles and stem throughout the winter and into late spring (Alford et al., 2003). There are three larval instars (Bonnemaison, 1965; Figure 1c–e). From late February to June, third instar larvae tunnel out of the plant, drop to the ground and create a small cavity a few centimetres under the soil surface to pupate (Williams & Carden, 1961; Figure 1f). Pupation lasts 8–12 weeks depending on temperature; new generation adults start to emerge in May within the OSR crop where they stay to feed on the stems and the exterior of pods (Sáringer, 1984; Williams & Carden, 1961; Figure 1g). In late summer, adults undergo a period of aestivation (prospective diapause; Sáringer, 1984) where they stop feeding and either remain in the crop (Sivcev et al., 2016; Vig, 2003) or migrate to sheltered areas such as hedgerows and woodlands (Bonnemaison & Jourdheuil, 1954; Figure 1h). By the end of August, when temperatures have cooled, the beetles become active again and migrate into newly sown crops, reaching the population peak by early September, although this varies with weather conditions (Sáringer, 1984; Vig, 2003).

### 1.2 Pest status

Both adult and larval stages of CSFB are damaging. Adults feed on cotyledons and young leaves of OSR plants giving rise to ‘shot‐holing’ symptoms (Figure 1a). Although in controlled conditions, plants can fully compensate for up to 90% leaf area loss at early growth stages (Coston, 2021; Ellis, 2015), damage to the hypocotyl at the cotyledon stage or severe and sustained feeding damage to the first leaves can threaten crop establishment. Once plants are beyond the four‐leaf stage, they are better able to compensate for leaf area loss and adult feeding damage becomes less important (Ruck et al., 2018). Larvae damage the plants by feeding (mining) within the petioles and stems (Williams & Carden, 1961; Figure 1c–e), causing reduced plant vigour and increased risk of frost damage and disease, reducing overwintering survival; in spring, they can cause stem splitting, death of the growing point, delayed flowering and even plant death (Bonnemaison & Jourdheuil, 1954; Evans, 2007; Williams & Carden, 1961). Traditionally, neonicotinoid seed treatments were the main method for protecting crops against adult CSFB (Maienfisch et al., 2001). However, since their withdrawal following concerns regarding their effects on non‐targets (Blacquière et al., 2012; Palmquist et al., 2012), pyrethroids are the only registered insecticidal control option but resistance is an increasing problem (Heimbach & Müller, 2012; Willis et al., 2020; Zimmer et al., 2014). The current situation reveals a threat to long‐term efficacy of insecticide use, making it necessary to have a broad range of management options available for farmers to combat CSFB in a sustainable and efficient way.

Current EU policy provides a framework for integrated pest management (IPM); defined by the Sustainable Use of Pesticides Directive (Directive 2009/128/EC), IPM offers ‘an approach to reduce the development of harmful organisms where plant protection products and methods are appropriately considered and kept to levels that are economically and ecologically justified and minimize risks to human health and the environment’. This Directive (Annex III) sets out a series of eight IPM principles (described by Barzman et al., 2015); namely: (1) Prevention and suppression of the pest through cultural actions; (2) pest monitoring – to enable (3) well‐judged decision‐making based on the actual and/or predicted pest incidence and specific thresholds. If an intervention is needed, IPM strategies offer a sequence of control options, giving preference to (4) sustainable biological, physical and other ‘non‐chemical’ methods. When insecticides are essential to provide control, (5) insecticide selection should favour selective products with fewest detrimental effects on the environment, non‐target organisms and human health. Also, IPM aims to (6) reduce insecticide use and (7) avoid insecticide resistance development. Principle 8 (evaluation) encourages users to evaluate the success of the actions and measures adopted to improve the process. Here we review the current evidence for existing components of IPM strategies to control CSFB in OSR following the structure defined by Directive 2009/128/EC and the principles described by Barzman et al. (2015) and highlight areas of research needed to improve them.

## 2 PRINCIPLE 1. PREVENTION AND SUPPRESSION VIA CULTURAL ACTIONS

### 2.1 Crop rotation

Crop rotation is used to prevent build‐up of pests, weeds and diseases and to maintain soil health; it is one of the fundamental aspects of IPM (AHDB, 2020). Initially, OSR was grown in c. one‐in‐five rotations with cereals (ENDURE, 2007). Longer rotations tend to result in increased yield (Zheng et al., 2020), but as the value of the crop has risen there has been a trend towards one‐in‐two or three‐year rotations (Berry & Spink, 2006; Rusch et al., 2010). The percentage of OSR in a region was shown to be negatively correlated with the proportion of plants with CSFB larvae or damage (Valantin‐Morison et al., 2007). However, this study was conducted several years before the peak in OSR production, and it is unknown if this ‘dilution effect’ applies to larger cropped areas and increased populations of beetles. Spatially, crops located close to the previous year's OSR seem to exhibit more damage than crops sown far from previous crops (Alves et al., 2015; Williams & Carden, 1961). However, because CSFB are highly mobile and can easily migrate between fields (Bonnemaison, 1965), crop rotation in itself is unlikely to disrupt their distribution unless done on a synchronized area‐wide basis (regionalized zoning) in which whole regions break from OSR cropping at the same time (Zheng et al., 2020).

### 2.2 Sowing date and seed‐bed conditions

Historically, the sowing window for a successful overwintering OSR crop was mid‐August to early September, although the optimum varies with latitude (Henke et al., 2009; Lääniste et al., 2007; Ratajczak et al., 2017; Williams & Carden, 1961). However, by sowing at this time, crop emergence coincides with CSFB immigration, making it susceptible to feeding attacks which can threaten establishment. Early sowing can enable crop establishment before CSFB migration, reducing crop vulnerability to adult CSFB (Alves et al., 2015; Valantin‐Morison et al., 2007; Wynn et al., 2017). However, early sowing can increase the risk of larval damage by lengthening the period available for CSFB oviposition (Conrad et al., 2021; White & Cowlrick, 2016). More research is required to understand the trade‐offs between crop establishment and larval damage and the interaction with timing of adult migration and establishment conditions.

Soil conditions at sowing are important. Crops drilled into light and fine soils with adequate moisture (40% by weight of water), particularly during emergence, establish quicker and are more able to withstand CSFB feeding damage (Alves et al., 2015; Blake et al., 2004; Wynn et al., 2017).

### 2.3 Cultivation method

There is a wide range of tillage regimes used for OSR, with differential effects on CSFB damage. Larval infestation is reduced when using minimum or zero tillage compared with ploughing (Ulber & Schierbaum‐Schickler, 2003; Valantin‐Morison et al., 2007). In reduced tillage systems, the presence of previous crop stubble, particularly tall stubble, reduces adult CSFB infestation (Ulber & Schierbaum‐Schickler, 2003; United Oilseeds, 2020). More work is needed to understand the mechanisms responsible for these observations.

Cultivation method can also impact the natural enemies of CSFB (see Section 5.1). Ground‐dwelling predators react differently to tillage method (Holland & Oakley, 2007) and reduced tillage has been found to increase their numbers in OSR (Büchs, 2003; Stinner & House, 1990; Thorbek & Bilde, 2004). Reduced tillage has been found to have positive effects on the abundance and survival of *Tersilochus migrogaster* Holmgren (Ichneumonidae: Tersilochinae) the main parasitoid of CSFB larvae (Ulber & Nitzsche, 2006). *T*. *microgaster* overwinters in diapause in the soil of former OSR fields (Ulber, Klukowski, et al., 2010), therefore, leaving fallow ground or using cultivation methods with minimal soil disturbance can reduce parasitoid mortality caused by ploughing (Nilsson, 2010).

### 2.4 Seed rate

Adult CSFB feeding is decreased at higher seed rates (Coston, 2021; White et al., 2020), likely due to dilution effects. Similarly, CSFB larval infestation per plant is significantly reduced with increasing plant density (Coston, 2021; Nuss & Ulber, 2004). However, final crop yield was not affected by seed rates (Coston, 2021; Nuss & Ulber, 2004; White et al., 2020). This is attributed to the ability of plants grown at low density to better compensate for larval damage and to produce larger petioles, more leaves and lateral racemes, providing enough food to avoid larval competition, thereby reducing migration to the main stem and terminal buds. However, by increasing OSR seed rate, total larvae/m^2^ could be increased; this may exacerbate problems in following seasons by increasing the total abundance of adult CSFB emerging from the crop (Nuss & Ulber, 2004; White et al., 2020).

### 2.5 Mowing/sheep grazing

The possibility of adapting canopy management techniques used for spring OSR crops such as livestock grazing (Syrovy et al., 2016) or mowing (Kirkegaard et al., 2008) for CSFB management has attracted recent interest. By removing OSR leaves infested with larvae, the number of third instar larvae entering stems and subsequent negative yield effects can be reduced. Winter OSR has been shown to compensate from defoliation with minimal impact on yield if occurring prior to stem elongation (Spink, 1992; Sprague et al., 2014). Later mowing led to a greater reduction in larval infestation compared to an unmown control: 31% in December, 42% in January and 55% in March (White et al., 2018). However, crops mown in March (close to stem elongation) had the lowest yields. Also, mowing the crop in early March delayed the onset of flowering which increased risk from pollen beetle (*Brassicogethes aeneus*) (Coston, 2021); this may have contributed to yield reductions recorded in comparison to unmown crops. In a farmer‐led study in the UK, larval numbers were significantly reduced in OSR crops when they were sheep‐grazed or mown (c. 75% and 45%, respectively); however, all defoliation resulted in yield loss compared with controls (Pickering & White, 2021). Further work is required to optimize timing and grazing intensity to overcome these negative impacts.

### 2.6 Companion planting

Two main companion planting approaches have been tested for CSFB: (1) sowing the crop with a ‘nurse crop’, that is, plants which protect the crop and are later removed after crop establishment and (2) trap cropping, where plants that are more attractive to the pest than the cash crop are grown alongside to divert pest pressure away from the cash crop (Cook et al., 2007; Hokkanen, 1991; Shelton & Badenes‐Pérez, 2006). The species used as ‘nurse plants’ are ideally fast growing but not highly competitive, frost sensitive and nitrogen providers. Tested species include faba beans, lentils, vetch, fenugreek, clovers, white mustard, buckwheat and nyger (Breitenmoser et al., 2020; Coston, 2021; Ruck et al., 2018). Reductions in CSFB adult damage and/or larval infestation have been reported when berseem clover was sown with OSR in France, Switzerland and UK (Breitenmoser et al., 2020; Verret et al., 2017; White et al., 2020; Seimandi‐Corda et al., unpublished data). The presence of cereal volunteers during establishment also reduced CSFB damage (Seimandi‐Corda et al. unpublished data). The approach ideally relies on die‐off of nurse plants in winter to avoid competition with the crop. However, in regions with mild winters like NW France and UK, removal with herbicide is required. This is problematic when OSR is combined with other Brassicas such as white mustard (*Sinapis alba*) and although use of ‘Clearfield’ cultivars resistant to specific herbicide overcome this, correct timing of removal of the nurse crop is difficult (Coston, 2021). Rigorous assessment of the efficacy of these practices is currently lacking.

Trap crops have shown potential to reduce CSFB infestation in OSR. In different field trials, OSR plots with turnip rape (*Brassica rapa*) borders were less damaged by adult CSFB (Coston, 2021) and had lower larval infestation (Barari et al., 2005; Coston, 2021) than plots without a trap crop. This is probably the result of the beetle's preference for turnip rape (Barari et al., 2005; Sivcev et al., 2016). Patches of volunteer OSR have also been shown to act as a trap crop reducing CSFB damage and larval infestation in OSR sown in close proximity (White et al., 2020). More research is needed to understand the mechanisms of action of nurse crops and trap crops and how implementation can be optimized by farmers.

### 2.7 Resistant cultivars

Although OSR cultivars resistant to several diseases have been successfully developed and are widely used, there are no insect‐resistant cultivars currently commercially available for any OSR pest (Hervé, 2017). Breeding plants with strong early vigour or good compensation mechanisms could increase plant tolerance to adult and larval infestation. Field data from commercial cultivars suggest that hybrids are generally more successful in withstanding CSFB pressure than conventional varieties, as they develop faster in autumn and/or spring, enabling them to grow away from adult and larval damage, respectively (Bayer, 2020; White et al., 2020).

The easiest way to develop resistant OSR cultivars is to identify resistant *B*. *napus* genotypes that can then be crossed with high‐yielding genotypes. Screening for reduced adult feeding has been conducted on a limited number of genotypes in the field or in controlled conditions (Åhman, 1993; Bartlet et al., 1996; Giamoustaris & Mithen, 1995; Lambdon et al., 1998) but no consistent differences were identified. More recent and ongoing research on larger OSR genotype sets seems to indicate some variability in resistance but results are inconsistent between laboratory and field trials (Cook et al., unpublished data; Thursfield et al., 2020). No differences in larval infestation between genotypes have been found (Döring & Ulber, 2020; White, 2016; White et al., 2020). However, mechanisms that confer insect resistance in other closely related *Brassica* species can also be used in OSR breeding programmes via introgression. Interspecific variability of CSFB adult feeding has been tested (Bartlet & Williams, 1991; Lambdon et al., 1998), and CSFB larvae have reduced weight and higher mortality when developing in white mustard compared to OSR (Döring & Ulber, 2020). Introgression of resistance to insects from this species to OSR has already been achieved (Gavloski et al., 2000; Kott & Dosdall, 2004) and could be possible for CSFB. Resistance mechanisms behind the intraspecific and interspecific variation observed remain largely unknown. Metabolites such as the glucosinolates (defence compounds specific to cruciferous plants including OSR) could be involved as these act as phagostimulants to CSFB adult feeding (Bartlet et al., 1994; Bartlet & Williams, 1991; Giamoustaris & Mithen, 1995) but contradictory results were found between feeding and glucosinolate levels (Bartlet et al., 1996, 1999).

Genetic modification enables OSR plants to express genes not usually found in the *Brassica* genome which confer resistance to insects (Hervé, 2017). Transformed OSR expressing the cysteine proteinase inhibitor (blocking protein digestion in insects) showed no effect on CSFB adults or larvae (Girard et al., 1998). In Canada, OSR transformed with *Arabidopsis thaliana* genes that induce the growth of dense trichomes at the cotyledon stage seems effective against *Phyllotreta* flea beetles and could also deter CSFB (Alahakoon, Adamson, et al., 2016; Alahakoon, Taheri, et al., 2016; Gruber et al., 2006; Soroka et al., 2011). Another potential approach is post‐transcriptional gene silencing via RNA interference (RNAi), which prevents the manufacture of key proteins in insects, leading to death when ingested (e.g. Baum et al., 2007). However, the EU currently has a restrictive regulation on the use of GM crops (Masip et al., 2013) limiting the adoption of such strategies.

## 3 PRINCIPLE 2. MONITORING

A key aspect of IPM programmes is assessing the risk of the crop suffering economically significant levels of damage. This assessment is usually based on the pests’ population density in the crop and/or direct assessment of injury levels via crop monitoring (scouting), and making use of scientifically‐based diagnosis and prediction systems when available (Barzman et al., 2015; Evans & Scarisbrick, 1994). As both adults and larval stages of CSFB are damaging, there are separate methods for monitoring and assessing the injury levels caused by each.

### 3.1 Monitoring adults

Yellow water traps are currently the main method of monitoring adult CSFB migration into newly sown OSR crops in autumn. The traps are placed at ground level in the crop and should be checked throughout CSFB immigration phase; the number of CSFB per trap is counted weekly (Walters & Lane, 1994). Although somewhat labour intensive for transportation of water and manual sorting (identifying and counting CSFB among by‐catch), these were found to be more effective than yellow sticky traps, which are often not sticky enough to trap adult CSFB (Green, 2008). Image‐based automatic identification applications, which make water trap assessments quicker and easier, are becoming commercially available (e.g. Xarvio scouting app which automatically counts and classifies insects including CSFB in the trap). Other image‐based sensor technologies are being developed to provide automatic identification of CSFB flight activity in real time (Hassall et al., 2021; Kirkeby et al., 2021). The use of attractant host plant volatiles (e.g. isothiocyanates [breakdown products of glucosinolates described above]; Bartlet et al., 1992) or sex/aggregation pheromones could improve monitoring efficacy. Male‐produced aggregation pheromones have been identified in *Phyllotreta* flea beetles (e.g. Beran et al., 2011; Peng & Weiss, 1992; Tóth et al., 2011). For CSFB, male‐specific antennal glands were discovered (Bartlet et al., 1994) suggesting that they may also secrete a sex pheromone, but this has not yet been chemically identified.

### 3.2 Monitoring larvae

Larval abundance is commonly assessed by dissecting OSR plants using a scalpel and counting the number of larvae found within the leaf petioles and stem (Walters & Lane, 1994). This method has been used to provide long‐term data on larval incidence (Crop Monitor, 2020; Nilsson, 2002). However, this is technically demanding and time‐consuming, and to do accurately it needs to be done using a binocular microscope. The larval evacuation method (Conrad et al., 2016) whereby field‐collected plants are left to dry in a container for 1–3 weeks stimulating larvae to naturally exit the plant takes less effort but is less accurate, as not all larvae may exit the plant. Furthermore, the delay between the samples being taken and when the farmer obtains the results does not enable timely control decisions. A third method for monitoring larval numbers is to count the percentage of leaves with scars on the petioles (Figure 1c–e; these characteristic marks are left as the larvae move between petioles for feeding), as there is a significant relationship between these and the number of larvae per plant during autumn (Walters et al., 2001). The number of larvae can be estimated from the number of adults in yellow water traps as these two factors are related (Green, 2008).

## 4 PRINCIPLE 3. DECISION FOR CONTROL BASED ON ACTION THRESHOLDS

Decision‐making regarding pest control in crop protection mainly involves using economic thresholds to decide whether or not there is a need to apply insecticide. Economic thresholds are defined as the lowest pest population density (pest per unit area, per plant or per part of plant) at which control measures are needed to prevent economic damage (Pedigo, 1986; Ramsden et al., 2017; Stern et al., 1959). The use of thresholds is critical to IPM as it allows farmers and agronomists to ensure that insecticides are only applied when necessary, that is, avoiding prophylactic use which may be unnecessary if the pest is not present at damaging levels. However, in most European countries (except for Switzerland; Ramseier et al., 2016), thresholds are for guidance only. There are no legal stipulations that oblige use of thresholds, which seems at odds with the EU directive that member states should put in place IPM strategies.

### 4.1 Reliability of action thresholds for CSFB control

For thresholds to be valuable, they must be based on scientific studies and consider the variation in crop damage, crop tolerance, control efficacy of the product as well as insecticide cost and crop value which are subject to varying market prices (Ellis & Berry, 2012; Ramsden et al., 2017). However, reviewing the current country‐specific thresholds for CSFB on the European continent (Table S1) indicates that peer‐reviewed empirical studies on the relationships between pest injury and yield validating such recommendations are uncommon and several countries may be using the same thresholds as neighbouring countries without validation. Godan (1950) first suggested the threshold of 5 CSFB larvae per plant as a threshold for treatment. Data on the equivalence between larval number per plant and yield loss are scarce but the threshold is clearly based on the economics of insecticide use rather than a physiological threshold above which plants are unable to compensate. In the UK, a threshold of 5 larvae/plant for CSFB was established based on the economics of organophosphates (Purvis, 1986). This was subsequently revised based on a lower return of £130/t (1991 World price), an average UK yield of 3 t/ha for OSR and cost of pyrethroids of £8/ha (Lane & Walters, 1993). This same threshold was revised again in 2007 as pyrethroids proved to be cost‐effective at 2 larvae/plant which provided an average yield response of 0.16 t/ha (HGCA, 2007) and reverted to 5 larvae/plant in 2013 to reflect lower efficacy due to pyrethroid resistance (AHDB‐HGCA, 2013). However, none of these studies showed the relationship between the number of larvae per plant and yield losses. To our knowledge, the amount of crop damage and/or yield loss caused per CSFB adult is still unknown. Mechanical damage in OSR (simulated injury) showed no effect on seed yield and percentage oil content but actual injury by *Phyllotreta* flea beetles led to significant reductions in both metrics (Antwi et al., 2008). Similarly, patch defoliation (akin to slug injury) and shot hole injury (akin to CSFB) led to differing compensatory responses in OSR, with patch defoliation showing full recovery and shot hole injury reducing seed grain yield compared to controls (Susko & Superfisky, 2009). In a recent study on the impact of simulated shot‐holing injury and controlled CSFB larval infestation, it was shown that OSR can compensate for leaf area injury of up to 90% at the cotyledon stage; however, significant reductions in plant height, yield and quality occurred when artificially infested with more than 5 CSFB larvae/plant (Coston, 2021). Further assessments are needed to quantify adult and larval damage, and their interaction, in field conditions to develop confidence in the use of thresholds.

Furthermore, considering the presence and abundance of natural enemies is an important, yet absent, component of economic thresholds, and has great potential for rationalizing insecticide use as they may increase the pest abundance level that a plant can tolerate before economic loss occurs.

### 4.2 Decision support systems (DSS)

The influence of weather factors on population dynamics of OSR pests, including CSFB, was studied in Germany; phenological models were developed and incorporated into a computer‐based DSS ‘proPlant’ (Johnen & Meier, 2000). This DSS allows the use of field observations (pest pressure, crop growth stage, growing conditions), combined with predicted local weather data to predict potential pest infestation, control requirements and optimal treatment dates (Johnen et al., 2010). The system included phenological models for CSFB which predicted immigration start, peaks of adults in the crop, start of oviposition and larval development, allowing more precise timing of monitoring and applications of insecticides targeted against adults to prevent oviposition and against larvae. The proPlant system was commercially used for CSFB control in mainland Europe (Johnen et al., 2010). The system is now part of the Xarvio Field Manager^®^ package but the CSFB model is not currently commercially available. Accurate models to predict adult CSFB migration (1–2 weeks in advance) could allow growers to better plan sowing dates to avoid peak migration or could be used to determine the need for seed treatment, as recently seen in the IPM strategy for sugar beet in UK (where emergency authorization is subject to the predicted level of virus yellows infection based on the migration date of the aphid vectors (Abram, 2021; Defra, 2021)). Understanding population cycles may also help to predict years when CSFB is a threat; long‐term monitoring data of larval populations in Sweden 1970–2000 suggest population peaks every 7–9 years (Nilsson, 2002), but such long‐term data are rare and it is unknown if these patterns are consistent across Europe and if they persisted on neonicotinoid treated crops to date.

## 5 PRINCIPLE 4. NON‐SYNTHETIC (NATURAL) CONTROL METHODS

If an intervention is needed, IPM strategies offer a sequence of control options to kill pests, giving preference to less environmentally damaging and sustainably produced ones. Barzman et al. (2015) terms these options ‘non‐chemical’ but we prefer the term ‘non‐synthetic’. These include biological, physical and a few other natural approaches.

### 5.1 Biological

Biological management of insect pests includes the use of live natural enemies in biocontrol and biopesticides. Understanding of the range of natural enemies of OSR pests and their impact has improved considerably over the last 20 years, mainly due to the completion of two EU‐funded research programmes investigating the potential for biocontrol in OSR (BORIS and MASTER; Alford, 2003; Williams, 2010b).

#### 5.1.1 Generalist predators

Three carabid (Coleoptera: Carabidae) species have been reported to be active and abundant in OSR at the time CSFB are migrating into new crops and oviposition starts: *Trechus quadristriatus* (Schrank) *Pterostichus madidus* (Fabricius) and *Nebria brevicollis* (Fabricius). Of these, *T*. *quadristriatus* and *P*. *madidus* showed significant spatial association with the larvae of CSFB during October but only *T*. *quadristriatus* fed on CSFB eggs in laboratory experiments (Warner et al., 2003). However, as there is no evidence that *T*. *quadristriatus* buries into the soil surface to feed, it is unclear whether CSFB eggs laid in the soil are accessible to them. Carabids could also feed on mature larvae leaving the plants to pupate in the soil (February–June). No information is available on which carabid species are most active in OSR during the early part of this period, but during May–June there are five species that could have biocontrol potential: *Amara similata* (Gyllenhal), *Anchomenus dorsalis* (Pontoppidan), *N*. *brevicollis*, *Asaphidion flavipes* (Linnaeus) and *Loricera pilicornis* (Fabricius) (Warner et al., 2008). Regarding spiders, money spiders (Linyphiidae) and wolf spiders (Lycosidae) are most abundant in OSR crops (Büchs & Alford, 2003; Nyffeler & Sunderland, 2003). However, there are no data on predation rates and effects on CSFB abundance by this group. There is therefore a need for more research to quantify the predation potential of generalist predators for conservation biocontrol of CSFB so that farmers can adopt appropriate habitat management measures to promote their populations.

#### 5.1.2 Specialist parasitoids

Within Europe, eight species of parasitic wasps (Hymenoptera) have been reported to target CSFB: six attack the larvae and two attack the adults (Jordan et al., 2020; Ulber, Klukowski, et al., 2010). The exact number is confused due to potential misidentifications as it is suggested that *Tersilochus tripartitus* (Brischke) resulted from misidentification of *T*. *microgaster* (Ulber, Klukowski, et al., 2010). *Tersilochus microgaster* (Szépligeti) has been reported to be the most abundant and frequently occurring parasitoid of larval‐stage CSFB in Europe (Barari et al., 2005; Klingenberg & Ulber, 1994; Nitzsche & Ulber, 1998; Ulber & Nitzsche, 2006; Ulber & Wedemeyer, 2004). The level of parasitism recorded for this species varies greatly, ranging from 40% to 50% in Germany (Döring et al., 2013; Ulber & Wedemeyer, 2004) to around 10% in UK (Barari et al., 2005; Ferguson et al., 2006). There is a close spatial association in OSR between larval stages of CSFB and *T*. *migrogaster* (Ferguson et al., 2006); such associations are necessary for effective biocontrol and indicate that the parasitoid is very efficient at finding its host and has good biocontrol potential. All the other larval parasitoid species appear to be of minor importance (Ulber & Williams, 2003). However, as larval parasitoids require full development of the larvae to complete their lifecycle they do not prevent economic damage, although they can help to reduce populations in the following year.

*Microctonus melanopus* (Ruthe) was long considered the only parasitoid attacking adult CSFB (Ulber & Williams, 2003; Ulber, Williams, et al., 2010), but in the past few years an additional species, *Microctonus brassicae* (Haeselbarth), has been identified and studied in the UK. Recent work on *M*. *brassicae* has described its lifecycle, behaviour and parasitism rate within captive colonies (Jordan et al., 2020; Ortega‐Ramos, 2021). Preliminary work indicates that parasitism rate in the field ranges from 0% to 36% in the UK (Ortega‐Ramos, 2021) and this species could represent an effective biocontrol agent either via conservation biological control or as part of augmentative releases. Further investigation on the lifecycle of *M*. *brassicae* in the field, its geographical distribution and the impacts of landscape and management factors on its populations are required to develop strategies to understand and improve its biocontrol potential.

### 5.2 Biopesticides

The term ‘biopesticide’ refers to a wide variety of pest management agents derived from natural (living) materials, that is, animals, plants and microorganisms (EPA, 2021). Biopesticides fall into three main classes: (1) entomopathogenic microorganisms (2) botanical pesticides and (3) animal‐derived pesticides.

Insect pathogens include entomopathogenic fungi (EPF), entomopathogenic nematodes (EPN), bacteria and protozoans; all occur naturally in most arable fields and play a vital role in insect population dynamics (Lacey et al., 2001). Entomopathogens already have a place in IPM programmes for some pests (Lacey et al., 2015; Maina et al., 2018). The most thoroughly studied EPFs for potential control of OSR pests are *Metarhizium anisopliae* and *Beauveria bassiana* and are known to infect adult CSFB; certain isolates when applied topically were reported to cause up to 88% and 40% mortality, respectively (Butt et al., 1992, 1994) and are currently being further tested against CSFB (Claire Hoarau, pers. com.; Pole, 2021). Both have strains with the capacity to colonize and grow endophytically inside OSR (Batta, 2013; Vidal & Jaber, 2015). Inoculation of plants via seed treatments could be a solution against CSFB but research is still in an early phase (reviewed by Card et al., 2015; Hokkanen & Menzler‐Hokkanen, 2017).

Entomopathogenic nematodes, particularly species of *Steinernema* have been found to be highly effective against most of the important OSR pests under field conditions (Hokkanen et al., 2006). In field studies conducted in UK and Sweden, *S*. *feltiae* reduced CSFB numbers by 73% and 60%, respectively (Hokkanen et al., 2006). Three *Steinernema* species, including *S*. *feltiae*, were tested along with *Heterorhabditis bacteriophora* against CSFB adults, with *H*. *bacteriophora* being most effective (Claire Horarau pers. com.) – this species has already been approved for control of black vine weevil (*Otiorhynchus sulcatus*; Pole, 2021).

Formulations derived from the bacteria *Bacillus thuringiensis* (Bt) are used widely as biopesticides against insect pests (Brar et al., 2006). Three formulations of Bt subspecies *tenebrionis* have been screened against CSFB but beetle mortality was low (Pole, 2021). Bt formulations against CSFB larvae are not considered of potential value due its production costs and difficulty in reaching the larval stages that live inside plant tissues (Evans & Scarisbrick, 1994). Regarding protozoans, none have been reported to attack CSFB.

Botanical insecticides are plant derivatives used to kill insects and can be applied as seed treatments or as sprays (Isman, 2006). Essential oil sprays have shown potential against some OSR pests (but not CSFB; Jiang et al., 2018; Pavela, 2011; Pavela et al., 2009); Azadirachtin, derived from the neem tree, was effective against *Phyllotreta* flea beetles (Reddy et al., 2014) but efficacy on CSFB has not been reported. The effect of 12 un‐named botanicals, seven biologicals and three promoters delivered as seed coatings was tested against adult CSFB; only one promotor significantly reduced feeding (by c. 50%) compared with the control (Lohaus et al., 2018); none were effective when applied topically onto the cotyledons.

FLiPPER^®^ is a natural by‐product of olive oil production and caused high mortality just 1 day after application in tests against CSFB (Pole, 2021). Another fatty‐acid‐based commercial product, M‐Pede^®^, was effective in trials against *Phyllotreta* flea beetles (Reddy et al., 2014) and could show promise against CSFB. Fatty acids can be derived from plants or animals and penetrate the insect cuticle to disrupt metabolic processes.

### 5.3 Physical and other non‐synthetic controls

Physical and mechanical methods used in pest management include trapping, barriers and physical destruction. Mass trapping, that is, the placement of several traps in the field to capture beetles seems impractical to manage CSFB in the absence of effective baits (sex/aggregation pheromones or attractive host plant volatiles). Various other non‐synthetic options such as silicates/rock‐dusts have been tested against OSR pests (Daniel et al., 2013; Faraone & Hillier, 2020) but not CSFB as far as we are aware.

## 6 PRINCIPLES 5–7. SYNTHETIC INSECTICIDES, INSECTICIDE SELECTION AND RESISTANCE MANAGEMENT

IPM approaches can include synthetic insecticides as a last resort for control; any insecticidal compounds should be ‘as specific as possible for the target and shall have the least side effects on human health, non‐target organisms and the environment’ (European Commission, 2021).

### 6.1 Neonicotinoids

Neonicotinoid insecticides came onto the market in 1991 and seed treatment formulations provided effective control against CSFB adults and early‐stage larvae for the first 6–8 weeks growth of the crop (Maienfisch et al., 2001; Sivcev et al., 2016). However, concerns over low levels found in nectar and consequent detrimental effects on honey bees (Blacquière et al., 2012; Palmquist et al., 2012) led the EU to restrict the use of clothianidin, thiamethoxam and imidacloprid neonicotinoids on seed and soil treatments on arable crops attractive to bees (including OSR) in December 2013 (European Commission, 2013). The restriction was revised in 2018 and the EU extended the ban on the three main neonicotinoids for all outdoor purposes (European Commission, 2018). After this, pyrethroids became the only method available for farmers to control CSFB.

### 6.2 Pyrethroids

Pyrethroid insecticides are synthetic forms of the botanical insecticide pyrethrin which is produced naturally by flowers of *Chrysanthemum cinerariifolium*. Developed in the mid‐1970s synthetic pyrethroids offered good control of a wide range of insect pests including CSFB (Soderlund, 2015). By 1990, pyrethroids replaced the more toxic and environmentally damaging organochlorines and organophosphates as foliar sprays, and continued to be used along with neonicotinoids to reduce egg‐laying by late‐season adults and to target newly emerged larvae and early instar larvae as they move between petioles (Zhang et al., 2017). Since the ban on neonicotinoid seed treatments, pyrethoid usage in OSR has increased drastically (FAOSTAT, 2021). Although farmers have been advised to use insecticides only when the pest exceeds the economic thresholds, pyrethroids are often applied several times in the same crop and sometimes prophylactically before the pest arrives (Defra, 2020; Williams, 2010a).

### 6.3 Alternative synthetic insecticides

In recent years, there have been several alternatives to pyrethroids trialled for CSFB management, with varying success. In 2013, Boravi WG (organophospate) was evaluated against CSFB and showed potential in controlling adults and larvae (Westerloppe, 2017). Methiocarb (carbamate) was used with emergency approval in 2014 in the UK on 9% of the OSR crop, but only 4% of agronomists reported differences in crop protection between treated and untreated seeds (Alves et al., 2015). Seed treatment with cyantraniliprole (DuPont Lumiposa^®^), a broad‐spectrum insecticide for use in OSR against CSFB and other autumn pests, is registered for use in OSR in Hungary, Poland and Romania with ongoing reviews in other Member States (NFU, 2018; Nieuwenhoven, 2017). Plots treated with Lumiposa had 65% less CSFB damage than untreated plots (Nieuwenhoven, 2017) but Coston (2021) found no significant effects.

### 6.4 Insecticide selection from an IPM perspective

IPM relies upon the application of selective insecticides that minimize unwanted effects on human health, non‐target organisms and the environment (Barzman et al., 2015). Although there are databases on pesticide selectivity that can be consulted (EPPO; Jansen, 2013), there is no formal procedure or practical guidance for insecticide selection. Biopesticides are generally understood as more environmentally friendly and safer than synthetic insecticides (Lacey et al., 2015; Lengai & Muthomi, 2018), but they are clearly not risk free. A key topic in the assessment of side effects is examining whether insecticides (synthetic or natural) affect beneficial or non‐target organisms. Some biopesticides, including natural pyrethrins, soft soap and mineral oils permitted in organic agriculture, are broad spectrum – as are some strains of entomopathogens (Bathon, 1996; Pavlyushin, 1996).

From an IPM perspective, neonicotinoid insecticidal seed treatments had many advantages when compared with other application methods especially sprays. Their systemic properties meant they could be applied to the seed prior to sowing, offering plant protection throughout the growing season without the need for repeated spray applications (Bass & Field, 2018). They only directly affect insects that feed on the plant, reducing contact and impact on non‐target organisms (Elbert et al., 2008), delivered control at a reduced rates (Tansey et al., 2008), and generally have less surface runoff (Palmquist et al., 2012) and reduced environmental concentration (Nuyttens et al., 2013) than sprays. However, many counterarguments have arisen against neonicotinoid use (Morrissey et al., 2015; Pisa et al., 2014); primarily that their systematic translocation to nectar and pollen negatively affects non‐target organisms, especially bees (reviewed by Blacquière et al., 2012; Lundin et al., 2015). It has also been argued that using insecticide‐treated seed is not compatible with IPM as it involves prophylactic application, before any assessment of pest abundance or crop damage (Bell, 2016). However, if the pest population can be forecasted, seed treatments could be used in specific cases when needed based on predictions (see DSS, Section 4.2).

Pyrethroid sprays, however, do not seem a good option form an IPM point of view. Pyrethroids are broad‐spectrum insecticides exhibiting very high toxicity to non‐target invertebrates including pollinators (Charreton et al., 2015; Sanchez‐Bayo & Goka, 2014), natural enemies (Desneux et al., 2004; Devotto et al., 2007; Douglas & Tooker, 2016) and aquatic invertebrates (e.g. Schulz et al., 2021). In OSR, pyrethroids are applied throughout the year to control different pests. Spring applications for pollen beetle control (Thieme et al., 2010) may coincide with activity of the CSFB larval parasitoid *T*. *microgaster* in the crop, negatively affecting their populations and biocontrol potential (Ulber, Williams, et al., 2010).

### 6.5 Insecticide use and resistance management

Resistance of insect pests to insecticides has long been an issue and was a major initial driver for the development of IPM (Stern et al., 1959). The increase in use of pyrethroid insecticides after the neonicotinoid ban has increased selection pressure and resulted in escalations in resistance in CSFB populations (Højland et al., 2015; Willis et al., 2020). The first confirmation of pyrethroid resistance in CSFB was in Germany, 2008 (Heimbach & Müller, 2012; Zimmer et al., 2014). Resistance was thereafter confirmed in UK (Foster & Williamson, 2015; Højland et al., 2015), Denmark (Højland & Kristensen, 2018), France (Bothorel et al., 2018; Robert et al., 2017) and Czech Republic (Stará & Kocourek, 2019). Three mechanisms conferring resistance have been discovered in CSFB: mutation in the voltage‐gate sodium channel conferring target‐site (knock‐down) resistance (kdr; Williamson et al., 1993); super‐knock down resistance (skdr), due to the L925I/M918L mutation (Bothorel et al., 2018; Willis et al., 2020) and metabolic‐based resistance (Foster & Williamson, 2015; Højland et al., 2015). Given how common and widespread resistance to pyrethroids is, it is reasonable to think that applications of this insecticidal class could be doing more harm than good; with reduced efficacy on the pest and reduced levels of control provided by affected natural enemies.

Insecticide resistance management strategies developed by the ‘Expert Committee on Pesticide Resistance – Insecticides’ (ECPR‐I, 2021) in Germany and the Insecticide Resistance Action Committee (IRAC, 2021) are available. Guidelines for resistance management include (1) correctly timing the applications; (2) use of products at recommended rates and (3) alternate different modes of actions. However, there are few other insecticides registered for CSFB that allow farmers to alternate products, making resistance management virtually impossible.

### 6.6 Reduced use

Reduced use of insecticides will, by the nature of resistance evolution, slow the development of insecticide resistance. Directive 2009/128/EC highlighted that ‘economic instruments can play a crucial role in the achievement of objectives relating to the sustainable use of pesticides’. Denmark has adopted a pesticide taxation linked to their environmental and health toxicity (Ministry of Environment & Food of Denmark, 2017; Pedersen et al., 2015). Norway, Sweden and France also have a pesticide tax (PAN, 2021) but these strategies do not necessarily result in reduced use reduce use (Böcker & Finger, 2016; Skevas et al., 2012). There is a clear need to provide farmers with solid, evidence‐based instructions and recommendations that they can follow to carry out IPM, perhaps supported by direct payments on ecological production (e.g. in Switzerland farmers receive payments based on agro‐ecological crop management (ENDURE, 2012).

## 7 PRINCIPLE 8. EVALUATION

Principle 8 encourages farmers to assess the soundness of the crop protection measures they adopt (Barzman et al., 2015), for example, by leaving control areas in each field; however, there are no definitive guidelines or processes by which farmers should evaluate their IPM strategy. Two new decision support initiatives are underway that may help to address this lack of holistic evaluation approaches for IPM in OSR (Munier‐Jolain & Paveley, 2021). Alternatively, evaluation strategies based on multicriteria analysis (Caffi et al., 2017) or long‐term studies using randomized controlled trials could be explored (Rejesus & Jones, 2020).

## 8 CONCLUSIONS

European agriculture is entering a future where fewer synthetic insecticides will be available and their use less profitable (due to reduced efficacy as a result of increased resistance in pest populations); consequently, pest management will need to rely on a wider range of methods, and this is particularly true for CSFB control in OSR. IPM strategies will be vital to providing a framework for sustainable pest management. However, this review highlights that a full IPM strategy for CSFB barely exists. We have analysed the gaps where more research is needed (Table 1) and hope this review will help identify those research and dissemination efforts that will bring adoption of IPM in OSR to its full potential.

**TABLE 1:** Summary of current measures available for use in integrated pest management strategies for cabbage stem flea beetle (CSFB) in oilseed rape (OSR), and main knowledge gaps for future research needed to improve measures

Principle	Current measures	Knowledge gaps
1. Prevention	Increased crop rotation, increased distance between previous years and current crop Sowing at the right time, with adequate moisture into a fine seed bed Use of minimum or zero tillage Companion cropping	Potential of synchronized regionalized zoning of OSR rotations Trade‐offs between crop establishment and larval damage and the interaction with timing of adult migration and establishment conditions Effect of stubble length to reduce immigration Timing of mowing/sheep grazing to reduce larval infestation Rigorous assessment of the efficacy of different nurse crop species and understanding of the mechanisms of action, and optimization of their agronomy Efficacy and spatial positioning of trap cropping; mechanisms of action Development of resistant or tolerant cultivars and understanding of mechanisms conferring protection.
2. Monitoring	Yellow water traps for monitoring adult CSFB Image‐based automatic identification applications for adults in yellow water traps Protocols for monitoring CSFB larval abundance via plant dissections, larval evacuation and counting plant scaring.	Sensor‐based automatic identification of adults in real time Identification, synthesis and formulation of attractant semiochemicals such as host plant volatiles or sex/aggregation pheromones
3. Decision‐making	Economic thresholds for adults and larval stages of CSFB Phenological model for egg laying and larval development	Defining a physiological threshold and understanding the relationship between the number of larvae/adults per plant and yield losses Quantification of the effect of natural enemies on pest population Phenological model for adult migration and prediction of abundance
4. Non‐synthetic (natural) control methods	Conservation biological control effected by natural enemies of CSFB	Quantification of the predation potential of generalist predators for conservation biocontrol of CSFB Data on geographical distribution of parasitoids and the impacts of landscape and agronomic management factors on their populations to develop strategies to use and improve their biocontrol potential. Identification of effective yet host‐specific strains of microorganisms, nematodes, protozoans and formulation as biopesticides Testing of botanical and other natural products for efficacy against CSFB Development of RNAi‐based formulations for spray‐induced gene silencing Identification of attractant baits (semiochemicals) for mass trapping
5. Synthetic insecticides Insecticide selection	Pyrethroid insecticides (spray application) New seed treatments: cyantraniliprole (DuPont Lumiposa^®^) European Plant Protection Agency regulation process	Development of highly specific insecticides targeted to CSFB with low environmental impact Protocols for grading or ranking insecticidal products according to target selectivity, detrimental effects on the environment, non‐target organisms and human health and production of clear guidance for farmers
6–7. Reduced use and Resistance management	Insecticide resistance management strategies developed by the ‘Expert Committee on Pesticide Resistance – Insecticides’ (ECPR‐I, 2021) in Germany and by Insecticide Resistance Action Committee (IRAC, 2021)	Updated strategies will be required once other insecticidal products are developed, registered and commercialized
8. Evaluation	Farmer evaluation method currently lacking. Other evaluation: European Parliamentary Research Service review of Directive 2009/128/EC (2018)	A framework/process whereby farmers can evaluate IPM methods and strategy outcomes – possibly as part of a decision support system, using multicriteria analysis or using randomized control trials in farmer field schools/cluster groups

## Supporting information

Table S1

## References

[gcbb12918-bib-0001] Abram, M. (2021). Neonic sugar beet derogation unlikely to be triggered. Farmers Weekly. https://www.fwi.co.uk/arable/sugar‐beet/neonic‐sugar‐beet‐derogation‐unlikely‐to‐be‐triggered

[gcbb12918-bib-0002] AHDB . (2020). Oilseed rape growth guide. https://ahdb.org.uk/osrgg

[gcbb12918-bib-0003] AHDB‐HGCA .(2013). Cabbage stem flea beetle. Information Sheet 24. May 2013.

[gcbb12918-bib-0004] Åhman, I. (1993). A search for resistance to insects in spring oilseed rape. IOBC‐WPRS Bulletin, 16, 36–46.

[gcbb12918-bib-0005] Alahakoon, U. , Adamson, J. , Grenkow, L. , Soroka, J. , Bonham‐Smith, P. , & Gruber, M. (2016). Field growth traits and insect‐host plant interactions of two transgenic canola (Brassicaceae) lines with elevated trichome numbers. The Canadian Entomologist, 148(05), 603–615. 10.4039/tce.2016.9

[gcbb12918-bib-0006] Alahakoon, U. I. , Taheri, A. , Nayidu, N. K. , Epp, D. , Yu, M. , Parkin, I. , Hegedus, D. , Bonham‐Smith, P. , & Gruber, M. Y. (2016). Hairy Canola (*Brasssica napus*) re‐visited: Down‐regulating TTG1 in an AtGL3‐enhanced hairy leaf background improves growth, leaf trichome coverage, and metabolite gene expression diversity. BMC Plant Biology, 16(1). 10.1186/s12870-015-0680-5 PMC4704247 26739276

[gcbb12918-bib-0007] Alford, D. V. (1979). Observations on the cabbage stem flea beetle, *Psylliodes chrysocephala*, on winter oil‐seed rape in Cambridgeshire. Annals of Applied Biology, 93, 117–123. 10.1111/j.1744-7348.1979.tb06521.x

[gcbb12918-bib-0008] Alford, D. V. (2003). Biocontrol of oilseed rape pests. Blackwell Science.

[gcbb12918-bib-0009] Alford, D. V. , Nilsson, C. , & Ulber, B. (2003). Insect pests of oilseed rape crops. In D. V. Alford (Ed.), Biocontrol of oilseed rape pests (pp. 9–42). Blackwell Science Ltd. https://onlinelibrary.wiley.com/doi/10.1002/9780470750988.ch2

[gcbb12918-bib-0010] Alves, L. , Wynn, S. & Stopps, J. (2015). Cabbage stem flea beetle live incidence and severity monitoring. AHDB Report, (551).

[gcbb12918-bib-0011] Andert, S. , Ziesemer, A. , & Zhang, H. (2021). Farmers’ perspectives of future management of winter oilseed rape (*Brassica napus* L.): A case study from north‐eastern Germany. European Journal of Agronomy, 130(126350). 10.1016/j.eja.2021.126350

[gcbb12918-bib-0012] Antwi, F. , Olson, D. L. , & DeVuyst, E. A. (2008). Growth responses of seedling canola to simulated versus *Phyllotreta cruciferae* (Coleoptera: Chrysomelidae) feeding injury to seedling canola. Journal of Entomological Science, 43(3), 320–330. 10.18474/0749-8004-43.3.320

[gcbb12918-bib-0013] Barari, H. , Ferguson, A. W. , Piper, R. W. , Smith, E. , Quicke, D. L. J. , & Williams, I. H. (2005). The separation of two hymenopteran parasitoids, *Tersilochus obscurator* and *Tersilochus microgaster* (Ichneumonidae), of stem‐mining pests of winter oilseed rape using DNA, morphometric and ecological data. Bulletin of Entomological Research, 95(04), 299–307. 10.1079/BER2005360 16048677

[gcbb12918-bib-0014] Bartlet, E. , Isidoro, N. , & Williams, I. H. (1994). Antennal glands in *Psylliodes chrysocephala*, and their possible role in reproductive behaviour. Physiological Entomology, 19, 241–250. 10.1111/j.1365-3032.1994.tb01048.x

[gcbb12918-bib-0015] Bartlet, E. , Kiddle, G. , Williams, I. , & Wallsgrove, R. (1999). Wound‐induced increases in the glucosinolate content of oilseed rape and their effect on subsequent herbivory by a crucifer specialist. Entomologia Experimentalis et Applicata, 91(1), 163–167. 10.1023/A:1003661626234

[gcbb12918-bib-0016] Bartlet, E. , Mithen, E. , & Clark, S. J. (1996). Feeding of the cabbage stem flea beetle *Psylloides chrysocephala* on high and low glucosinolate cultivars of oilseed rape. Entomologia Experimentalis Et Applicata, 80, 87–89.

[gcbb12918-bib-0017] Bartlet, E. , Parsons, D. , Williams, I. H. , & Clark, S. J. (1994). The influence of glucosinolates and sugars on feeding by the cabbage stem flea beetle, *Psylliodes chrysocephala* . Entomologia Experimentalis et Applicata, 73, 77–83. 10.1111/j.1570-7458.1994.tb01841.x

[gcbb12918-bib-0019] Bartlet , & Williams, I. H. (1991). Factors restricting the feeding of the cabbage stem flea beetle (*Psylliodes chrysocephala*). Entomologia Experimentalis et Applicata, 60(3), 233–238. 10.1111/j.1570-7458.1991.tb01543.x

[gcbb12918-bib-1003] Bartlet, E. , Williams, I. H. , Blight, M. M. , & Hick, A. J. (1992). Response of the oilseed rape pests, *Ceutorhynchus assimilis* and *Psylliodes chrysocephala*, to a mixture of isothiocyanates. In Proceedings of the 8th international symposium on insect‐plant relationships (pp. 103–104). Springer.

[gcbb12918-bib-0020] Barzman, M. , Bàrberi, P. , Birch, A. N. E. , Boonekamp, P. , Dachbrodt‐Saaydeh, S. , Graf, B. , Hommel, B. , Jensen, J. E. , Kiss, J. , Kudsk, P. , Lamichhane, J. R. , Messéan, A. , Moonen, A. C. , Ratnadass, A. , Ricci, P. , Sarah, J. L. , & Sattin, M. (2015). Eight principles of integrated pest management. Agronomy for Sustainable Development, 35(4), 1199–1215. 10.1007/s13593-015-0327-9

[gcbb12918-bib-0022] Bass, C. , & Field, L. M. (2018). Neonicotinoids. Current Biology, 28(14), R772–R773. 10.1016/j.cub.2018.05.061 30040932

[gcbb12918-bib-0023] Batta, Y. (2013). Efficacy of endophytic and applied *Metarhizium anisopliae* (Metch.) Sorokin (Ascomycota: Hypocreales) against larvae of *Plutella xylostella* L. (Yponomeutidae: Lepidoptera) infesting *Brassica napus* plants. Crop Protection, 44, 128–134.

[gcbb12918-bib-1015] Bathon, H. (1996). Impact of entomopathogenic nematodes on non‐target hosts. Biocontrol Science and Technology, 6, 421–434. 10.1080/09583159631398

[gcbb12918-bib-0024] Baum, J. A. , Bogaert, T. , Clinton, W. , Heck, G. R. , Feldmann, P. , Ilagan, O. , Johnson, S. , Plaetinck, G. , Munyikwa, T. , Pleau, M. , & Vaughn, T. (2007). Control of coleopteran insect pests through RNA interference. Nature Biotechnology, 25, 1322–1326. 10.1038/nbt1359 17982443

[gcbb12918-bib-0025] Bell, S. (2016). Farming oilseed rape without neonicotinoids. Friends of the Earth, 33.

[gcbb12918-bib-0026] Beran, F. , Mewis, I. , Srinivasan, R. , Svoboda, J. , Vial, C. , Mosimann, H. , Boland, W. , Büttner, C. , Ulrichs, C. , Hansson, B. S. , & Reinecke, A. (2011). Male *Phyllotreta striolata* (F.) produce an aggregation pheromone: Identification of male‐specific compounds and interaction with host plant volatiles. Journal of Chemical Ecology, 37(1), 85–97. 10.1007/s10886-010-9899-7 21181241

[gcbb12918-bib-0027] Berry, P. M. , & Spink, J. H. (2006). A physiological analysis of oilseed rape yields: Past and future. Journal of Agricultural Science, 144(5), 381–392. 10.1017/S0021859606006423

[gcbb12918-bib-0028] Blacquière, T. , Smagghe, G. , Van Gestel, C. A. M. , & Mommaerts, V. (2012). Neonicotinoids in bees: A review on concentrations, side‐effects and risk assessment. Ecotoxicology, 21(4), 973–992. 10.1007/s10646-012-0863-x 22350105 PMC3338325

[gcbb12918-bib-0029] Blake, J.J. , Spink, J.H. & Bullard, M.J. (2004). Successful establishment of oilseed rape. HGCA conference: Managing soil and roots for profitable production.

[gcbb12918-bib-0030] Böcker, T. , & Finger, R. (2016). European pesticide tax schemes in comparison: An analysis of experiences and developments. Sustainability, 8(4), 378. 10.3390/su8040378

[gcbb12918-bib-0031] Bonnemaison, L. (1965). Insect pests of crucifers and their control. Annual Review of Entomology, 10(1), 233–256. 10.1146/annurev.en.10.010165.001313

[gcbb12918-bib-0032] Bonnemaison, L. , & Jourdheuil, P. (1954). L’altise d’hiver du colza (*Psylliodes chrysocephala* L.). Ann Épiphyties, 4, 345–524.

[gcbb12918-bib-0033] Bothorel, S. , Robert, C. , Ruck, L. , Carpezat, J. , Lauvernay, A. , Leflon, M. , & Siegwart, M. (2018). Resistance to pyrethroid insecticides in cabbage stem flea beetle (*Psylliodes chrysocephala*) and rape winter stem weevil (*Ceutorhynchus picitarsis*) populations in France. Integrated Control in Oilseed Crops IOBC‐WPRS Bulletin, 136, 89–104.

[gcbb12918-bib-0034] Brar, S. K. , Verma, M. , Tyagi, R. D. , & Valéro, J. R. (2006). Recent advances in downstream processing and formulations of *Bacillus thuringiensis* based biopesticides. Process Biochemistry, 41, 323–342. 10.1016/j.procbio.2005.07.015

[gcbb12918-bib-0035] Breitenmoser, S. , Steinger, T. , Hiltpold, I. , Grosjean, Y. , Nussbaum, V. , Bussereau, F. , & Baux, A. (2020). Effet des plantes associées au colza d’hiver sur les dégâts d’altises. Rech Agron Suisse, 11, 16–25.

[gcbb12918-bib-0036] Büchs, W. (2003). Impact of on‐farm landscape structures and farming systems on predators. In D. V. Alford (Ed.), Biocontrol of oilseed rape pests (pp. 254–278). Blackwell.

[gcbb12918-bib-0037] Büchs, W. , & Alford, D. (2003). Predators or oilseed rape pests. In D. V. Alford (Ed.), Biocontrol of oilseed rape (pp. 181–199). Blackwell Science Ltd.

[gcbb12918-bib-0039] Butt, T. M. , Barrisever, M. , Drummond, J. , Schuler, T. H. , Tillemans, F. T. , & Wilding, N. (1992). Pathogenicity of the entomogenous, hyphomycete fungus, *Metarhizium anisopliae* against the chrysomelid beetles *Psylliodes chrysocephala* and *Phaedon cochleariae* . Biocontrol Science and Technology, 2(4), 327–334. 10.1080/09583159209355248

[gcbb12918-bib-0040] Butt, T. M. , Ibrahim, L. , Ball, B. V. , & Clark, S. J. (1994). Pathogenicity of the entomogenous fungi *Metarhizium anisopliae* and *Beauveria bassiana* against crucifer pests and the honey bee. Biocontrol Science and Technology, 4(2), 207–214. 10.1080/09583159409355328

[gcbb12918-bib-0042] Caffi, T. , Helsen, H. H. M. , Rossi, V. , Holb, I. J. , Strassemeyer, J. , Buurma, J. S. , Capowiez, Y. , Simon, S. , & Alaphilippe, A. (2017). Multicriteria evaluation of innovative IPM systems in pome fruit in Europe. Crop Protection, 97, 101–108. 10.1016/j.cropro.2016.12.009

[gcbb12918-bib-1008] Card, S. D. , Hume, D. E. , Roodi, D. , McGill, C. R. , Millner, J. P. , & Johnson, R. D. (2015). Beneficial endophytic microorganisms of Brassica—A review. Biological Control, 90, 102–112. 10.1016/j.biocontrol.2015.06.001

[gcbb12918-bib-0043] Charreton, M. , Decourtye, A. , Henry, M. , Rodet, G. , Sandoz, J. C. , Charnet, P. , & Collet, C. (2015). A locomotor deficit induced by sublethal doses of pyrethroid and neonicotinoid insecticides in the honeybee *Apis mellifera* . PLoS One, 10(12), 1–14. 10.1371/journal.pone.0144879 PMC4682844 26659095

[gcbb12918-bib-0044] Conrad, N. , Brandes, M. , & Heimbach, U. (2016). Automatic extraction of *Psylliodes chrysocephala* larvae versus sorting by hand. IOBC‐WPRS Bulletin, 116, 63–66.

[gcbb12918-bib-0045] Conrad, N. , Brandes, M. , Ulber, B. , & Heimbach, U. (2021). Effect of immigration time and beetle density on development of the cabbage stem flea beetle, (*Psylliodes chrysocephala* L.) and damage potential in winter oilseed rape. Journal of Plant Diseases and Protection, 128(4), 1081–1090. 10.1007/s41348-021-00474-7

[gcbb12918-bib-0046] Cook, S. M. , Khan, Z. R. , & Pickett, J. A. (2007). The use of push‐pull strategies in integrated pest management. Annual Review of Entomology, 52(1), 375–400. 10.1146/annurev.ento.52.110405.091407 16968206

[gcbb12918-bib-0047] Coston, D. J. (2021). Quantifying the impacts of the neonicotinoid restriction on oilseed rape pest control and productivity. PhD thesis, University of Reading

[gcbb12918-bib-0048] Crop Monitor . (2020). 2019/2020 survey: Autumn pest assessment 2019. https://secure.fera.defra.gov.uk/cropmonitor/wosr/surveys/wosrPestAssLab.cfm

[gcbb12918-bib-0049] Daniel, C. , Dierauer, H. , & Clerc, M. (2013). The potential of silicate rock dust to control pollen beetles (*Meligethes* spp.). IOBC‐WPRS Bulletin, 96, 47–55.

[gcbb12918-bib-1000] Defra . (2017). Farming Statistics Provisional crop areas, yields and livestock populations At June 2017‐United Kingdom. https://www.gov.uk/government/statistics/farming‐statistics‐provisional‐crop‐areas‐yields‐and‐livestock‐populations‐at‐1‐june‐2017‐united‐kingdom

[gcbb12918-bib-0050] Defra . (2020). Farming statistics—Provisional arable crop areas at 1 June 2020, England. https://assets.publishing.service.gov.uk/government/uploads/system/uploads/attachment_data/file/910585/structure‐jun2020provcrops‐eng‐20aug20.pdf

[gcbb12918-bib-0051] Defra . (2021). Statement on the decision to issue – with strict conditions – emergency authorisation to use a product containing a neonicotinoid to treat sugar beet seed in 2021. https://www.gov.uk/government/publications/neonicotinoid‐product‐as‐seed‐treatment‐for‐sugar‐beet‐emergency‐authorisation‐application/statement‐on‐the‐decision‐to‐issue‐with‐strict‐conditions‐emergency‐authorisation‐to‐use‐a‐product‐containing‐a‐neonicotinoid‐to‐treat‐sugar‐beet

[gcbb12918-bib-0052] Desneux, N. , Pham‐Delègue, M. H. , & Kaiser, L. (2004). Effects of sub‐lethal and lethal doses of lambda‐cyhalothrin on oviposition experience and host‐searching behaviour of a parasitic wasp, *Aphidius ervi* . Pest Management Science, 60(4), 381–389. 10.1002/ps.822 15119601

[gcbb12918-bib-0053] Devotto, L. , Carrillo, R. , Cisternas, E. , & Gerding, M. (2007). Effects of lambda‐cyhalothrin and *Beauveria bassiana* spores on abundance of Chilean soil surface predators, especially spiders and carabid beetles. Pedobiologia, 51(1), 65–73. 10.1016/j.pedobi.2007.01.001

[gcbb12918-bib-0054] Döring, A. , Mennerich, D. & Ulber, B. (2014). Parasitism of cabbage stem flea beetle in oilseed rape and turnip rape. IOBC‐WPRS Bulletin 104: 41

[gcbb12918-bib-0055] Döring, A. , & Ulber, B. (2020). Performance of cabbage stem flea beetle larvae (*Psylliodes chrysocephala*) in brassicaceous plants and the effect of glucosinolate profiles. Entomologia Experimentalis et Applicata, 1–9. 10.1111/eea.12891

[gcbb12918-bib-0056] Douglas, M. R. , & Tooker, J. F. (2016). Meta‐analysis reveals that seed‐applied neonicotinoids and pyrethroids have similar negative effects on abundance of arthropod natural enemies. PeerJ, 4(12), e2776. 10.7717/peerj.2776 27957400 PMC5147019

[gcbb12918-bib-0057] Ebbe‐Nyman, E. (1952). Rapsjordloppan *Psylliodes chrysocephala* L. bidrag till Kannedom om dess biologi och bekiimpning. Statens Viixtskyddsanstalt Meddelande, 63, 1–103.

[gcbb12918-bib-0058] ECPR‐I . (2021). Fachausschuss Insektizide / Akarizide: Resistenzstrategien. https://www.julius‐kuehn.de/pflanzenschutz/fachausschuesse‐pflanzenschutzmittelresistenz/

[gcbb12918-bib-0060] Elbert, A. , Haas, M. , Springer, B. , Thielert, W. , & Nauen, R. (2008). Applied aspects of neonicotinoid uses in crop protection. Pest Management Science, 64(11) ), 1099–1105. 10.1002/ps.1616 18561166

[gcbb12918-bib-0061] Ellis, S. (2015). Project Report No . 546 Maximising control of cabbage stem flea beetles (CSFB) without neonicotinoid seed treatments. AHDB Report (546).

[gcbb12918-bib-0062] Ellis, S. A. , & Berry, P. M. (2012). Re‐evaluating thresholds for pollen beetle in oilseed rape. HGCA Project Report No. 495.

[gcbb12918-bib-0063] ENDURE . (2007). Report on RA2.6a—Designing Innovative crop protection strategies in arable rotations—Winter Crops Based Cropping Systems (WCCS): Design of AS and IS1 cropping systems.

[gcbb12918-bib-0064] ENDURE . (2012). Country profile: Switzerland. http://www.endure‐network.eu/de/about_endure/all_the_news/country_profile_switzerland

[gcbb12918-bib-0065] EPA . (2021). What are biopesticides? https://www.epa.gov/ingredients‐used‐pesticide‐products/what‐are‐biopesticides

[gcbb12918-bib-0067] European Commission . (2013). Commission Implementing Regulation (EU) No. 485/2013 of 24 May. Official Journal of the European Union, 56, 12–26. 10.2903/j.efsa.2013.3067

[gcbb12918-bib-0068] European Commission . (2018). Oilseeds and protein crops market situation—Committee for the common organisation of agricultural markets (February). https://ec.europa.eu/agriculture/sites/agriculture/files/cereals/presentations/cereals‐oilseeds/market‐situation‐oilseeds_en.pdf

[gcbb12918-bib-1010] European Commission . (2021). Integrated Pest Management (IPM). https://ec.europa.eu/food/plants/pesticides/sustainable‐use‐pesticides/integrated‐pest‐management‐ipm_en

[gcbb12918-bib-0070] European Parliamentary Research Service . (2018). European implementation Assessment of Directive 2009/128/EC on the sustainable use of pesticides. M. Remáč (Ed.). https://www.europarl.europa.eu/RegData/etudes/STUD/2018/627113/EPRS_STU(2018)627113_EN.pdf

[gcbb12918-bib-0071] Evans, A. (2007). Stem boring pests of winter oilseed rape. Technical Note. The Scottish Agriculture College, January, 3–6.

[gcbb12918-bib-0072] Evans, K. A. , & Scarisbrick, D. H. (1994). Integrated insect pest management in oilseed rape crops in Europe. Crop Protection, 13(6), 403–412. 10.1016/0261-2194(94)90086-8

[gcbb12918-bib-0073] Faostats . (2021). Food and Agriculture Organization of the United Nations. (1997). FAOSTAT statistical database. FAO.

[gcbb12918-bib-0074] Faraone, N. , & Hillier, N. K. (2020). Preliminary evaluation of a granite rock dust product for pest herbivore management in field conditions. Insects, 11, 1–11. 10.3390/insects11120877 PMC7763347 33322278

[gcbb12918-bib-0075] Ferguson, A. W. , Barari, H. , Warner, D. J. , Campbell, J. M. , Smith, E. T. , Watts, N. P. , & Williams, I. H. (2006). Distributions and interactions of the stem miners *Psylliodes chrysocephala* and *Ceutorhynchus pallidactylus* and their parasitoids in a crop of winter oilseed rape (*Brassica napus*). Entomologia Experimentalis et Applicata, 119(2), 81–92. 10.1111/j.1570-7458.2006.00404.x

[gcbb12918-bib-0076] Foster, S. , & Williamson, M. (2015). Investigating pyrethroid resistance in UK cabbage stem flea beetle populations and developing a PCR‐based assay for detecting turnip yellows virus in aphids. AHDB Report (55).

[gcbb12918-bib-0077] Furth, D. G. (1988). The jumping apparatus of flea beetles (Alticinae) — The metafemoral spring. In P. Jolivet , E. Petitpierre , & T. H. Hsiao (Eds.), Biology of Chrysomelidae. Series Entomologica (Vol. 42, pp. 285–297). Springer.

[gcbb12918-bib-0078] Gavloski, J. E. , Ekuere, U. , Keddie, A. , Dosdall, L. , Kott, L. , & Good, A. G. (2000). Identification and evaluation of flea beetle (*Phyllotreta cruciferae*) resistance within Brassicaceae. Canadian Journal of Plant Science, 80(4), 881–887.

[gcbb12918-bib-0079] Giamoustaris, A. , & Mithen, R. (1995). The effect of modifying the glucosinolate content of leaves of oilseed rape (*Brassica napus* ssp. *oleifera*) on its interaction with specialist and generalist pests. Annals of Applied Biology, 126(2), 347–363. 10.1111/j.1744-7348.1995.tb05371.x

[gcbb12918-bib-0080] Girard, C. , Le Métayer, M. , Zaccomer, B. , Bartlet, E. , Williams, I. , Bonadé‐Bottino, M. , Pham‐Delegue, M. H. , & Jouanin, L. (1998). Growth stimulation of beetle larva reared on a transgenic oilseed rape expressing a cysteine proteinase inhibitor. Journal of Insect Physiology, 44(3–4), 263–270. 10.1016/S0022-1910(97)00142-X 12769960

[gcbb12918-bib-0081] Godan, D. (1950). Wann ist der Rapserdflohlarven‐Befall für den Rapsacker gefährlicher, im Herbst oder im Frühjahr? Nachrichtenblatt des Deutschen Pflanzenschutzdienstes, 2, 149–153.

[gcbb12918-bib-0082] Green, D. B. (2008). Revised Thresholds for Cabbage Stem Flea Beetle on Oilseed Rape, HGCA Project report 428. HGCA, UK.

[gcbb12918-bib-0083] Gruber, M. Y. , Wang, S. , Ethier, S. , Holowachuk, J. , Bonham‐Smith, P. C. , Soroka, J. , & Lloyd, A. (2006). “HAIRY CANOLA”—Arabidopsis GL3 induces a dense covering of trichomes on *Brassica napus* seedlings. Plant Molecular Biology, 60(5), 679–698. 10.1007/s11103-005-5472-0 16649106

[gcbb12918-bib-0084] Hassall, K. L. , Dye, A. , Potamitis, I. , & Bell, J. R. (2021). Resolving the identification of weak‐flying insects during flight: A coupling between rigorous data processing and biology. Agricultural and Forest Entomology, 23(4), 489–505. 10.1111/afe.12453 34819800 PMC8596709

[gcbb12918-bib-0085] Heimbach, U. , & Müller, A. (2012). Incidence of pyrethroid‐resistant oilseed rape pests in Germany. Pest Management Science, 69(2), 209–216. 10.1002/ps.3351 23345124

[gcbb12918-bib-0086] Henke, J. , Sieling, K. , Sauermann, W. , & Kage, H. (2009). Analysing soil and canopy factors affecting optimum nitrogen fertilization rates of oilseed rape (*Brassica napus*). The Journal of Agricultural Science, 147(1), 1–8.

[gcbb12918-bib-0087] Hervé, M. R. (2018). Breeding for insect resistance in oilseed rape: Challenges, current knowledge and perspectives. Plant Breeding, 137(1), 27–34. 10.1111/pbr.12552

[gcbb12918-bib-0088] HGCA . (2007). Revised thresholds for economic cabbage stem flea beetle control. Topic Sheet 98 / Summer 2007. http://adlib.everysite.co.uk/adlib/defra/content.aspx?doc=248519&id=248521

[gcbb12918-bib-0089] Højland, D. H. , & Kristensen, M. (2018). Target‐site and metabolic resistance against λ‐cyhalothrin in cabbage stem flea beetles in Denmark. Bulletin of Insectology, 71(1), 45–49.

[gcbb12918-bib-0090] Højland, D. H. , Nauen, R. , Foster, S. P. , Williamson, M. S. , & Kristensen, M. (2015). Incidence, spread and mechanisms of pyrethroid resistance in European populations of the cabbage stem flea beetle, *Psylliodes chrysocephala* L. (Coleoptera: Chrysomelidae). PLoS One, 10(12). 10.1371/journal.pone.0146045 PMC4696833 26717570

[gcbb12918-bib-0091] Hokkanen, H. M. T. (1991). Trap cropping in pest management. Annual Review of Entomology, 36(80), 119–138. 10.1146/annurev.en.36.010191.001003

[gcbb12918-bib-0092] Hokkanen, H. M. T. , & Menzler‐Hokkanen, I. M. (2017). Use of entomopathogenic fungi in the insect pest management of Brassica oilseed crops. In G. V. P. Reddy (Ed.), Integrated management of insect pests on canola and other Brassica oilseed crops (pp. 373–382). CABI. 10.1079/9781780648200.0373

[gcbb12918-bib-0093] Hokkanen, H. M. T. , Vojinovic, M. Z. , Husberg, G.‐B. , Menzler‐Hokkanen, B. W. , Klukowski, Z. , Luik, A. , Nilsson, C. , Ulber, B. , & Williams, I. (2006). Effectiveness of entomopathogenic nematodes in the control of OSR pests. CD Proc Int Symp Integrated Pest Management in Oilseed Rape, 3–5 April 2006, Goettingen, Germany.

[gcbb12918-bib-0094] Holland, J. M. , & Oakley, J. (2007). Importance of arthropod pests and their natural enemies in relation to recent farming practice changes in the UK. HGCA Res Rev.

[gcbb12918-bib-0096] IRAC . (2021). Resistance basis: Management. https://irac‐online.org/about/resistance/management/

[gcbb12918-bib-0097] Isman, M. B. (2006). Botanical insecticides, deterrents, and repellents in modern agriculture and an increasingly regulated world. Annual Review of Entomology, 51(1), 45–66. 10.1146/annurev.ento.51.110104.151146 16332203

[gcbb12918-bib-1012] Jansen, J. (2013). Pest select database: A new tool to use selective pesticides for IPM. Communications in Agricultural and Applied Biological Sciences, 78, 115–119. 25145231

[gcbb12918-bib-0098] Jiang, H. , Wang, J. , Song, L. I. , Cao, X. , Yao, X. I. , Tang, F. , & Yue, Y. (2018). Chemical composition of an insecticidal extract from *Robinia pseudacacia* L. seeds and it's efficacy against aphids in oilseed rape. Crop Protection, 104, 1–6. 10.1016/j.cropro.2017.10.004

[gcbb12918-bib-0099] Johnen, A. , & Meier, H. (2000). A weather‐based decision support system for managing oilseed rape pests, 1–3(May), 793–800.

[gcbb12918-bib-0100] Johnen, A. , Williams, I. H. , Nilsson, C. , Klukowski, Z. , Luik, A. , & Ulber, B. (2010). The proPlant decision support system: Phenological models for the major pests of oilseed rape and their key parasitoids in Europe. In Biocontrol‐based integrated management of oilseed rape pests (pp. 381–403). Springer. 10.1007/978-90-481-3983-5_15

[gcbb12918-bib-0101] Jordan, A. , Broad, G. R. , Stigenberg, J. , Hughes, J. , Stone, J. , Bedford, I. , Pen, S. , & Wells, R. (2020). The potential of the solitary parasitoid *Microctonus brassicae* for the biological control of the adult cabbage stem flea beetle. Psylliodes Chrysocephala. Entomologia Experimentalis et Applicata, 1–11. 10.1111/eea.12910 PMC7386932 32742005

[gcbb12918-bib-0102] Kirkeby, C. , Rydhmer, K. , Cook, S. M. , Strand, A. , Torrance, M. T. , Swain, J. L. , Prangsma, J. , Johnen, A. , Jensen, M. , Brydegaard, M. , & Græsbøll, K. (2021). Advances in automatic identification of flying insects using optical sensors and machine learning. Scientific Reports, 11(1), 1–8. 10.1038/s41598-021-81005-0 33452353 PMC7810676

[gcbb12918-bib-0103] Kirkegaard, J. A. , Sprague, S. J. , Dove, H. , Kelman, W. M. , Marcroft, S. J. , Lieschke, A. , Howe, G. N. , & Graham, J. M. (2008). Dual‐purpose canola—A new opportunity in mixed farming systems. Australian Journal of Agricultural Research, 59, 291–302. 10.1071/AR07285

[gcbb12918-bib-0104] Klingenberg, A. , & Ulber, B. (1994). Untersuchungen zum Auftreten der Tersilochinae (Hym., Ichneumonidae) als Larvalparasitoide einiger Raps‐schädlinge im Raum Göttingen 1990 und 1991 und zu deren Schlupfabundanz nach unterschiedlicher Bodenbearbeitung. Journal of Applied Entomology, 117(1–5), 287–299. 10.1111/j.1439-0418.1994.tb00737.x

[gcbb12918-bib-0105] Kott, L. S. , & Dosdall, L. M. (2004). Introgression of root maggot resistance (Delia spp.) derived from *Sinapis alba* L. into *Brassica napus* L. Brassica, 6, 55–62.

[gcbb12918-bib-0106] Lääniste, P. , Jõudu, J. , Eremeev, V. , & mäeorg, E. (2007). Sowing date influence on winter oilseed rape overwintering in Estonia. Acta Agriculturae Scandinavica Section B‐Soil and Plant Science, 57(4), 342–348. 10.1080/09064710601029554

[gcbb12918-bib-0107] Lacey, L. A. , Frutos, R. , Kaya, H. K. , & Vail, P. (2001). Insect pathogens as biological control agents: Do they have a future? Biological Control, 21(3), 230–248. 10.1006/bcon.2001.0938

[gcbb12918-bib-0108] Lacey, L. A. , Grzywacz, D. , Shapiro‐Ilan, D. I. , Frutos, R. , Brownbridge, M. , & Goettel, M. S. (2015). Insect pathogens as biological control agents: Back to the future. Journal of Invertebrate Pathology, 132, 1–41. 10.1016/j.jip.2015.07.009 26225455

[gcbb12918-bib-0109] Lambdon, P. W. , Hassall, M. , & Mithen, R. (1998). Feeding preferences of woodpigeons and flea‐beetles for oilseed rape and turnip rape. Annals of Applied Biology, 133(3), 313–328. 10.1111/j.1744-7348.1998.tb05833.x

[gcbb12918-bib-1006] Lane, A. , & Walters, K. F. A. (1993). Recent incidence and cost effective control of pests of oilseed rape in England and Wales. In: Bulletin OILB SROP (France).

[gcbb12918-bib-0110] Lengai, G. M. W. , & Muthomi, J. W. (2018). Biopesticides and their role in sustainable agricultural production. Journal of Biosciences and Medicines, 06(06), 7–41. 10.4236/jbm.2018.66002

[gcbb12918-bib-0111] Lohaus, K. , Köneke, A. S. , Feussner, K. , Zienkiewicz, K. , & Ulber, B. (2018). Efficacy of alternative seed coatings against autumn insect pests of winter oilseed rape (*Brassica napus* L.). Integrated Control in Oilseed Crops IOBC‐WPRS Bulletin, 136, 113).

[gcbb12918-bib-0112] Lundin, O. , Rundlöf, M. , Smith, H. G. , Fries, I. , & Bommarco, R. (2015). Neonicotinoid insecticides and their impacts on bees: A systematic review of research approaches and identification of knowledge gaps. PLoS One, 10(8), 1–20. 10.1371/journal.pone.0136928 PMC4552548 26313444

[gcbb12918-bib-0113] Maienfisch, P. , Angst, M. , Brandl, F. , Fischer, W. , Hofer, D. , Kayser, H. , Kobel, W. , Rindlisbacher, A. , Senn, R. , Steinemann, A. , & Widmer, H. (2001). Chemistry and biology of thiamethoxam: A second generation neonicotinoid. Pest Management Science, 57(10), 906–913. 10.1002/ps.365 11695183

[gcbb12918-bib-0114] Maina, U. , Galadima, I. B. , Gambo, F. M. , & Zakaria, D. (2018). A review on the use of entomopathogenic fungi in the management of insect pests of field crops. Journal of Entomology and Zoology Studies, 6(1), 27–32.

[gcbb12918-bib-0115] Masip, G. , Sabalza, M. , Pérez‐Massot, E. , Banakar, R. , Cebrian, D. , Twyman, R. , Capell, T. , Albajes, R. , & Christou, P. (2013). Paradoxical EU agricultural policies on genetically engineered crops. Trends in Plant Science, 18, 312–324. 10.1016/j.tplants.2013.03.004 23623240

[gcbb12918-bib-0116] Mathiasen, H. , Bligaard, J. , & Esbjerg, P. (2015). Survival of cabbage stem flea beetle larvae, *Psylliodes chrysocephala*, exposed to low temperatures. Entomologia Experimentalis et Applicata, 157(2), 220–226.

[gcbb12918-bib-0117] Mathiasen, H. , Sørensen, H. , Bligaard, J. , & Esbjerg, P. (2015). Effect of temperature on reproduction and embryonic development of the cabbage stem flea beetle, *Psylliodes chrysocephala* L., (Coleoptera: Chrysomelidae). Journal of Applied Entomology, 139(8), 600–608. 10.1111/jen.12201

[gcbb12918-bib-0118] Meuche, A. (1940). Untersuchungen am Rapserdfloh (*Psylliodes chrysocephala* L.) in Ostholstein. Zeitschrift Für Angewandte Entomologie, 27, 464–495. 10.1111/j.1439-0418.1940.tb00501.x

[gcbb12918-bib-0119] Ministry of environment and food of Denmark . (2017). Danish national action plan on pesticides 2017–2021. https://ec.europa.eu/food/sites/food/files/plant/docs/pesticides_sup_nap_dan‐rev_en.pdf

[gcbb12918-bib-1014] Morrissey, C. A. , Mineau, P. , Devries, J. H. , Sanchez‐Bayo, F. , Liess, M. , Cavallaro, M. C. , & Liber, K. (2015). Neonicotinoid contamination of global surface waters and associated risk to aquatic invertebrates: A review. Environment International, 74, 291–303. 10.1016/j.envint.2014.10.024 25454246

[gcbb12918-bib-0120] Munier‐Jolain, N. , & Paveley, N. (2021). Integrated pest management: Reducing pesticide use in European farms. https://www.openaccessgovernment.org/integrated‐pest‐management/112485/

[gcbb12918-bib-0121] NFU . (2018). Lumiposa treated oilseed rape seed. https://www.nfuonline.com/cross‐sector/science‐and‐technology/crop‐protection/crop‐protection/lumiposa‐treated‐oilseed‐rape‐seed/

[gcbb12918-bib-0122] Nieuwenhoven, A. (2017). Cyantraniliprole insecticide seed treatment: A new and unique tool for integrated pest management in oilseed rape in Europe Anita. EPPO Workshop on Integrated Management of Insect Pests in Oilseed Rape EPPO Workshop.

[gcbb12918-bib-0123] Nilsson, C. (2002). Strategies for the control of cabbage stem flea beetle on winter rape in Sweden. Integrated Control in Oilseed Crops IOBC/WPRS Bulletin, 25, 133–139.

[gcbb12918-bib-0124] Nilsson, C. (2010). Impact of soil tillage on parasitoids of oilseed rape pests. In I. H. Williams (Ed.), Biocontrol‐based integrated management of oilseed rape pests (pp. 305–311). 10.1007/978-90-481-3983-5_11

[gcbb12918-bib-0125] Nitzsche, O. , & Ulber, B. (1998). Influence of different tillage treatments following the harvest of oilseed‐rape on the mortality of pollen beetle (Meligethes spp.) parasitoids. Zeitschrift Für Pflanzenkrankheiten Und Pflanzenschutz /. Journal of Plant Diseases and Protection, 105(4), 417–421.

[gcbb12918-bib-0126] Nuss, H. , & Ulber, B. (2004). Effect of sowing density of oilseed rape on the abundance and within‐plant distribution of cabbage stem flea beetle, *Psylliodes chrysocephala* . International Organisation for Biological Control Bulletin, 27(10), 223.

[gcbb12918-bib-0127] Nuyttens, D. , Devarrewaere, W. , Verboven, P. , & Foqué, D. (2013). Pesticide‐laden dust emission and drift from treated seeds during seed drilling: A review. Pest Management Science, 69(5), 564–575. 10.1002/ps.3485 23456984

[gcbb12918-bib-0128] Nyffeler, M. , & Sunderland, K. D. (2003). Composition, abundance and pest control potential of spider communities in agroecosystems: A comparison of European and US studies. Agriculture, Ecosystems and Environment, 95(2–3), 579–612. 10.1016/S0167-8809(02)00181-0

[gcbb12918-bib-0129] Ortega‐Ramos, P. A. (2021). Ecology and distribution of cabbage stem flea beetle and its parasitoids in UK winter oilseed rape: Steps towards integrated pest management. University of Reading, 221 pp.

[gcbb12918-bib-0130] Palmquist, K. , Salatas, J. , & Fairbrother, A. (2012). Pyrethroid insecticides: Use, environmental fate, and ecotoxicology. In Insecticides—Advances in integrated pest management (pp. 251–278). 10.5772/29495

[gcbb12918-bib-0131] PAN . (2021). Pesticide taxaction. https://www.pan‐europe.info/issues/pesticide‐taxation

[gcbb12918-bib-0132] Pavela, R. (2011). Insecticidal and repellent activity of selected essential oils against of the pollen beetle, *Meligethes aeneus* (Fabricius) adults. Industrial Crops and Products, 34(1), 888–892. 10.1016/j.indcrop.2011.02.014

[gcbb12918-bib-0133] Pavela, R. , Kazda, J. , & Herda, G. (2009). Effectiveness of Neem (*Azadirachta indica*) insecticides against Brassica pod midge (*Dasineura brassicae* Winn.). Journal of Pest Science, 82(3), 235–240. 10.1007/s10340-009-0244-2

[gcbb12918-bib-1016] Pavlyushin, V. A. (1996). Effect of entomopathogenic fungi on entomophagous arthropods. IOBC/WPRS Bulletin, 19, 247–249.

[gcbb12918-bib-0134] Pedersen, A. B. , Nielsen, H. Ø. , & Andersen, M. S. (2015). The Danish pesticide tax. In M. Lago , J. Mysiak , C. Gómez , G. Delacámara , & A. Maziotis (Eds.), Use of economic instruments in water policy. Global issues in water policy (Vol. 14). Springer. 10.1007/978-3-319-18287-2_6

[gcbb12918-bib-0135] Pedigo, L. (1986). Economic injury levels in theory and practice. Annual Review of Entomology, 31(1), 341–368. 10.1146/annurev.ento.31.1.341

[gcbb12918-bib-0136] Peng, C. , & Weiss, M. J. (1992). Evidence of an aggregation pheromone in the flea beetle, *Phyllotreta cruciferae* (Gocze) (Coleoptera: Chrysomelidae). Journal of Chemical Ecology, 18, 875–884. 24254091 10.1007/BF00988328

[gcbb12918-bib-0137] Pickering, F. , & White, S. (2021) Defoliation of winter oil seed rape for cabbage stem flea beetle management 19/20. Final report of the Innovative Farmers Field Lab. https://www.innovativefarmers.org/field‐lab?id=1312df59‐6747‐ea11‐817e‐005056ad0bd4

[gcbb12918-bib-1019] Pisa, L. W. , Amaral‐Rogers, V. , Belzunces, L. P. , Bonmatin, J. M. , Downs, C. A. , Goulson, D. , Kreutzweiser, D. P. , Krupke, C. , Liess, M. , McField, M. , Morrissey, C. A. , Noome, D. A. , Settele, J. , Simon‐Delso, N. , Stark, J. D. , Van der Sluijs, J. P. , Van Dyck, H. , & Wiemers, M. (2014). Effects of neonicotinoids and fipronil on non‐target invertebrates. Environmental Science and Pollution Research, 22, 68–102. 10.1007/s11356-014-3471-x 25223353 PMC4284392

[gcbb12918-bib-0139] Pole, J. (2021). Students’ union: Beetles bashed by biologicals. Arable Focus AHDB. https://projectblue.blob.core.windows.net/media/Default/ImportedPublicationDocs/AHDBCereals&Oilseeds/ArableFocus/2021/ArableFocus_MAY2021_WEBV3.pdf

[gcbb12918-bib-1005] Purvis, G. (1986). The influence of cabbage stem flea beetle (*Psylliodes chrysocephela* (L.)) on yields of oilseed rape. British Crop Protection Conference – Pests and Diseases, 2, 753–759.

[gcbb12918-bib-0140] Ramsden, M. W. , Kendall, S. L. , Ellis, S. A. , & Berry, P. M. (2017). A review of economic thresholds for invertebrate pests in UK arable crops. Crop Protection, 96, 30–43. 10.1016/j.cropro.2017.01.009

[gcbb12918-bib-1004] Ramseier, H. , Lebrun, M. , & Steinger, T. (2016). Use of economic damage thresholds, forecasting systems and warning services in Switzerland. https://www.agrarforschungschweiz.ch/en/2016/02/use‐of‐economic‐damage‐thresholds‐forecasting‐systems‐and‐warning‐services‐in‐switzerland/

[gcbb12918-bib-0141] Ratajczak, K. , Sulewska, H. , & Szymańska, G. (2017). New winter oilseed rape varieties–seed quality and morphological traits depending on sowing date and rate. Plant Production Science, 20(3), 262–272. 10.1080/1343943X.2017.1304809

[gcbb12918-bib-0142] Reddy, G. V. P. , Tangtrakulwanich, K. , Wu, J. , Miller, O. H. , Ophus, V. L. , & Prewett, J. (2014). Sustainable management tactics for control of *Phyllotreta cruciferae* (Coleoptera: Chrysomelidae) on canola in Montana. Entomological Society of America, 107, 661–666. 10.1016/j.cropro.2014.08.013 24772547

[gcbb12918-bib-0143] Rejesus, R. M. , & Jones, M. S. (2020). Perspective: Enhancing economic evaluations and impacts of integrated pest management Farmer Field Schools (IPM‐FFS) in low‐income countries. Pest Management Science, 76(11), 3527–3536. 32418359 10.1002/ps.5912

[gcbb12918-bib-0144] Robert, C. , Ruck, L. , Carpezat, J. , Lauvernay, A. , Siegwart, M. & Leflon, M. (2017). Suivi des resistances des populations d’altises d’hiver (psylliodes chrysocephala) et de charançon du bourgeon terminal (ceutorhynchus picitarsis) aux pyrethrinoïdes en france en culture de colza. AFPP – 11e Conférence Internationale Sur Les Ravageurs et Auxiliaires En Agriculture Montpellier – 25 et 26 Octobre.

[gcbb12918-bib-0145] Ruan, Y. , Konstantinov, A. S. , Shi, G. , Tao, Y. , Li, Y. , Johnson, A. J. , & Yang, X. (2020). The jumping mechanism of flea beetles (Coleoptera, chrysomelidae, alticini), its application to bionics and preliminary design for a robotic jumping leg. ZooKeys, 2020, 87–105. 10.3897/zookeys.915.38348 PMC7052025 32148424

[gcbb12918-bib-0146] Ruck, L. , Cadoux, S. , & Robert, C. (2018). Agronomic practices to control cabbage stem flea beetle and rape winter stem weevil. Integrated Control in Oilseed Crops: IOBC‐WPRS Bulletin, 65‐67.

[gcbb12918-bib-0147] Rusch, A. , Valantin‐Morison, M. , Sarthou, J. , & Roger‐Estrade, J. (2010). Integrating crop and landscape management into new crop protection strategies to enhance biological control of oilseed rape pests. In I. I. H. Williams (Ed.), Biocontrol‐based integrated management of oilseed rape pests (pp. 415–448). Springer.

[gcbb12918-bib-0148] Sanchez‐Bayo, F. , & Goka, K. (2014). Pesticide residues and bees—A risk assessment. PLoS One, 9(4). 10.1371/journal.pone.0094482 PMC3981812 24718419

[gcbb12918-bib-0149] Sáringer, G. (1984). Summer diapause of cabbage stem flea beetle, *Psylliodes chrysocephala* L. (Col. Chrysomelidae). Zeitschrift Für Angewandte Entomologie, 98(1–5), 50–54. 10.1111/j.1439-0418.1984.tb02683.x

[gcbb12918-bib-0150] Schulz, R. , Bub, S. , Petschick, L. L. , Stehle, S. , & Wolfram, J. (2021). Applied pesticide toxicity shifts toward plants and invertebrates, even in GM crops. Science, 372(6537), 81–84. 33795455 10.1126/science.abe1148

[gcbb12918-bib-0151] Shelton, A. , & Badenes‐Pérez, F. (2006). Concepts and applications of trap cropping in pest management. Annual Review of Entomology, 51, 285–308. 10.1146/annurev.ento.51.110104.150959 16332213

[gcbb12918-bib-0152] Sivcev, L. , Graora, D. , Sivcev, I. , Tomic, V. , & Dudic, B. (2016). Phenology of cabbage stem flea beetle (*Psylliodes chrysocephala* L.) in oilseed rape. Pesticidi i Fitomedicina, 31(3–4), 139–144. 10.2298/PIF1604139S

[gcbb12918-bib-0153] Skevas, T. , Stefanou, S. E. , & Lansink, A. O. (2012). Can economic incentives encourage actual reductions in pesticide use and environmental spillovers? Agricultural Economics, 43(3), 267–276. 10.1111/j.1574-0862.2012.00581.x

[gcbb12918-bib-0154] Soderlund, D. M. (2015). Resmethrin, the first modern pyrethroid insecticide. Pest Management Science, 71(6), 801–807. 10.1002/ps.3881 25124081

[gcbb12918-bib-0155] Soroka, J. J. , Holowachuk, J. M. , Gruber, M. Y. , & Grenkow, L. F. (2011). Feeding by flea beetles (Coleoptera: Chrysomelidae; Phyllotreta spp.) is decreased on canola (*Brassica napus*) seedlings with increased trichome density. Journal of Economic Entomology, 104(1), 125–136. 10.1603/EC10151 21404849

[gcbb12918-bib-0156] Spink, J. H. (1992). Winter oilseed rape: Effect of partial defoliation. HGCA Project Report, n. S4.

[gcbb12918-bib-0157] Sprague, S. J. , Kirkegaard, J. A. , Graham, J. M. , Dove, H. , & Kelman, W. M. (2014). Crop and livestock production for dual‐purpose winter canola (*Brassica napus*) in the high‐rainfall zone of south‐eastern Australia. Field Crops Research, 156, 30–39. 10.1016/j.fcr.2013.10.010

[gcbb12918-bib-0158] Stará, J. , & Kocourek, F. (2019). Cabbage stem flea beetle’s (*Psylliodes chrysocephala* L.) susceptibility to pyrethroids and tolerance to thiacloprid in the Czech Republic. PLoS One, 14(9), e0214702. 10.1371/journal.pone.0214702 31539393 PMC6754130

[gcbb12918-bib-0159] Stern, V. R. , Smith, R. , van den Bosch, R. , & Hagen, K. (1959). The integration of chemical and biological control of the spotted alfalfa aphid: The integrated control concept. Hilgardia, 29, 81–101. 10.1192/bjp.111.479.1009-a

[gcbb12918-bib-0160] Stinner, B. , & House, G. (1990). Arthropods and other invertebrates in conservation‐tillafe agriculture. Annual Review of Entomology, 35, 299–318.

[gcbb12918-bib-0161] Susko, D. J. , & Superfisky, B. (2009). A comparison of artificial defoliation techniques using canola (*Brassica napus*). Plant Ecology, 202, 169–175. 10.1007/s11258-008-9462-6

[gcbb12918-bib-0162] Syrovy, L. D. , Shirtliffe, S. J. , & Zarnstorff, M. E. (2016). Yield response to early defoliation in spring‐planted canola. Crop Science, 56, 1981–1987. 10.2135/cropsci2015.09.0556

[gcbb12918-bib-0163] Tansey, J. A. , Dosdall, L. M. , & Keddie, B. A. (2008). *Phyllotreta cruciferae* and *Phyllotreta striolata* responses to insecticidal seed treatments with different modes of action. Journal of Applied Entomology, 133(3), 201–209. 10.1111/j.1439-0418.2008.01321.x

[gcbb12918-bib-0165] Thieme, T. , Heimbach, U. , & Müller, A. (2010). Chemical control of insect pests and insecticide resistance in oilseed rape. In I. H. Williams (Ed.), Biocontrol‐based integrated management of oilseed rape pests (pp. 313–336). Springer.

[gcbb12918-bib-0166] Thorbek, P. , & Bilde, T. (2004). Reduced numbers of generalist arthropod predators after crop management. Journal of Applied Ecology, 41(3), 526–538. 10.1111/j.0021-8901.2004.00913.x

[gcbb12918-bib-0167] Thursfield, L. , Wells, R. , Faure, S. , & Penfield, S. (2020). Finding novel pest resistance to the cabbage stem flea beetle in oilseed rape. BCPC Pest and Beneficials Meeting.

[gcbb12918-bib-0168] Tóth, M. , Csonka, É. , Bartelt, R. J. , Cossé, A. A. , & Zilkowski, B. W. (2011). Similarities in pheromonal communication of flea beetles *Phyllotreta cruciferae* Goeze and Ph. vittula Redtenbacher (Coleoptera, Chrysomelidae). Journal of Applied Entomology, 136(9), 688–697. 10.1111/j.1439-0418.2011.01702.x

[gcbb12918-bib-0169] Ulber, B. , Klukowski, Z. , & Williams, I. (2010). Impact of insecticides on parasitoids of oilseed rape. In E. H. Williams (Ed.), Biocontrol‐based integrated management of oilseed rape pests (pp. 337–356). Springer.

[gcbb12918-bib-0170] Ulber, B. , & Nitzsche, O. (2006). Phenology of parasitoids (Hym., Ichneumonidae‐Tersilochinae) of oilseed rape pests in northern Germany from 1995‐1997. Integrated Control in Oilseed Crops IOBC/Wprs Bulletin, 29(7), 173–179.

[gcbb12918-bib-0171] Ulber, B. , & Schierbaum‐Schickler, C. (2003). The effect of tillage regime on the infestation of oilseed rape by the cabbage stem flea beetle, *Psylliodes chrysocephala* . 11th International Rapeseed Congress, Vol. 3, Copenhagen, 1037.

[gcbb12918-bib-0172] Ulber, B. , & Wedemeyer, R. (2004). Incidence of larval parasitism of *Psylliodes chrysocephala* within oilseed rape crops in Germany. IOBC/Wprs Bulletin 2, 7(10), 273–278.

[gcbb12918-bib-0173] Ulber, B. , & Williams, I. H. (2003). Parasitoids of flea beetles. In D. V. Alford (Ed.), Biocontrol of oilseed rape pests (pp. 125–144). Blackwell Science.

[gcbb12918-bib-0174] Ulber, B. , Williams, I. H. , Klukowski, Z. , Luik, A. , & Nilsson, C. (2010). Parasitoids of oilseed rape pests in Europe: Key species for conservation biocontrol. In I. H. Willliams (Ed.), Biocontrol‐based integrated management of oilseed rape pests (pp. 45–76). Springer.

[gcbb12918-bib-0175] United Oilseeds . (2020). Reducing the impact of CSFB. https://www.unitedoilseeds.co.uk/grow‐your‐crop

[gcbb12918-bib-0176] USDA . (2019). EU‐28 biofuels annual EU biofuels annual 2019. https://apps.fas.usda.gov/newgainapi/api/report/downloadreportbyfilename?filename=BiofuelsAnnual_TheHague_EU‐28_7‐15‐2019pdf

[gcbb12918-bib-0177] USDA . (2020). Oilseeds and products annual. https://apps.fas.usda.gov/newgainapi/api/Report/DownloadReportByFileName?fileName=OilseedsandProductsAnnual_Vienna_EuropeanUnion_04‐01‐2020

[gcbb12918-bib-0178] Valantin‐Morison, M. , Meynard, J. M. , & Doré, T. (2007). Effects of crop management and surrounding field environment on insect incidence in organic winter oilseed rape (*Brassica napus* L.). Crop Protection, 26(8), 1108–1120. 10.1016/j.cropro.2006.10.005

[gcbb12918-bib-0179] Verret, V. , Gardarin, A. , Makowski, D. , Lorin, M. , Cadoux, S. , Butier, A. , & Valantin‐Morison, M. (2017). Assessment of the benefits of frost‐sensitive companion plants in winter rapeseed. European Journal of Agronomy, 91, 93–103.

[gcbb12918-bib-0180] Vidal, S. , & Jaber, L. R. (2015). Entomopathogenic fungi as endophytes: Plant–endophyte–herbivore interactions and prospects for use in biological control. Current Science, 46–54.

[gcbb12918-bib-0181] Vig, K. (2003). Data on the biology of cabbage stem flea beetle, *Psylliodes chrysocephalus* (Linnaeus, 1758) (Coleoptera, Chrysomelidae, Alticinae). Communications in Agricultural and Applied Biological Sciences, 68(September), 231–237. 15149113

[gcbb12918-bib-0182] Walters, K. F. A. , & Lane, A. (1994). Sampling procedures for pests of winter oilseed rape: Meeting the needs of the crop consultant. Aspects of Applied Biology, 37(83).

[gcbb12918-bib-0183] Walters, K. F. A. , Lane, A. , Cooper, D. A. , & Morgan, D. (2001). A commercially acceptable assessment technique for improved control of cabbage stem flea beetle feeding on winter oilseed rape. Crop Protection, 20(10), 907–912. 10.1016/S0261-2194(01)00040-0

[gcbb12918-bib-0185] Warner, D. J. , Allen‐Williams, L. J. , Warrington, S. , Ferguson, A. W. , & Williams, I. H. (2003). Mapping, characterisation, and comparison of the spatio‐temporal distributions of cabbage stem flea beetle (*Psylliodes chrysocephala*), carabids, and Collembola in a crop of winter oilseed rape (*Brassica napus*). Entomologia Experimentalis et Applicata, 109(3), 225–234. 10.1046/j.0013-8703.2003.00112.x

[gcbb12918-bib-0186] Warner, D. J. , Allen‐Williams, L. J. , Warrington, S. , Ferguson, A. W. , & Williams, I. H. (2008). Implications for conservation biocontrol of spatio‐temporal relationships between carabid beetles and coleopterous pests in winter oilseed rape. Agricultural and Forest Entomology, 10(4), 375–387. 10.1111/j.1461-9563.2008.00391.x

[gcbb12918-bib-0187] Westerloppe, L. (2017). A tool of interest for the management of oilseed rape’s Cabbage Stem Flea Beetles resistant to pyrethroids. Écologie Chimique: Nouvelles Contributions à La Protection Des Cultures Contre Les Ravageurs et 11e Conférence Internationale Sur Les Ravageurs et Auxiliaires En Agriculture, 24 Au 26 Octobre 2017. Association Française de Protection.

[gcbb12918-bib-0188] White, S. (2016). Cabbage stem flea beetle larval survey (2015). AHDB Cereals & Oilseed Publications, (January), 19. https://cereals.ahdb.org.uk/media/876041/214‐0025‐annual‐project‐report‐2015.pdf

[gcbb12918-bib-1002] White, S. , & Cowlrick, S. (2016). Project report no. PR586. Cabbage stem flea beetle larval survey. AHDB Cereal Oilseeds PR586:14, https://projectblue.blob.core.windows.net/media/Default/Research%20Papers/Cereals%20and%20Oilseed/cabbage‐stem‐flea‐beetle‐larval‐survey‐2016‐.pdf

[gcbb12918-bib-0189] White, S. , Ellis, S. , & Kendall, S. (2018). Investigating non‐chemical control options for cabbage stem flea beetle in oilseed rape. Integrated Control in Oilseed Crops: IOBC‐WPRS Bulletin.

[gcbb12918-bib-0190] White, S. , Ellis, S. , Pickering, F. , Leybourne, D. , Corkley, I. , Kendall, S. , Collins, L. , Newbert, M. , Cotton, L. , & Phillips, R. (2020). Project Report No. 623 Integrated pest management of cabbage stem flea beetle in oilseed rape. AHDB Cereals and Oilseeds (623).

[gcbb12918-bib-0191] Williams, I. H. (2010a). Biocontrol‐based integrated management of oilseed rape pests. Biocontrol‐Based Integrated Management of Oilseed Rape Pests. 10.1007/978-90-481-3983-5

[gcbb12918-bib-0192] Williams, I. H. (2010b). The major insect pests of oilseed rape in europe and their management: An overview. In Biocontrol‐based integrated management of oilseed rape pests (pp. 1–43). Springer. 10.1007/978-90-481-3983-5_1

[gcbb12918-bib-0193] Williams, J. J. W. , & Carden, P. W. (1961). Cabbage stem flea beetle in east Anglia. Plant Pathology, 10(3), 85–95. 10.1111/j.1365-3059.1961.tb00124.x

[gcbb12918-bib-0194] Williamson, M. , Denholm, I. , Bell, C. A. , & Devonshire, A. L. (1993). Knockdown resistance (kdr) to DDT and pyre‐ throid insecticides maps to a sodium channel gene locus in the house fly (*Musca domestica*). Molecular and General Genetics, 240, 17–22. 8101963 10.1007/BF00276878

[gcbb12918-bib-0195] Willis, C. E. , Foster, S. P. , Zimmer, C. T. , Elias, J. , Chang, X. , Field, L. M. , Williamson, M. S. , & Davies, T. G. E. (2020). Investigating the status of pyrethroid resistance in UK populations of the cabbage stem flea beetle (*Psylliodes chrysocephala*). Crop Protection, 138, 105316. 10.1016/j.cropro.2020.105316 33273750 PMC7607605

[gcbb12918-bib-0196] Wynn, S. , Ecclestone, E. , Carter, R. , & Certer, R. (2017). Cabbage stem flea beetle live incidence and severity monitoring Autumn 2016 and Spring 2017. ADHB Cereals & Oilseeds Reports (571).

[gcbb12918-bib-0198] Zhang, H. , Breeze, T. , Bailey, A. , Garthwaite, D. , Harrington, R. , & Potts, S. G. (2017). Arthropod pest control for UK oilseed rape—Comparing insecticide efficacies, side effects and alternatives. PLoS One, 12(1). 10.1371/journal.pone.0169475 PMC5226783 28076392

[gcbb12918-bib-0199] Zheng, X. , Koopmann, B. , Ulber, B. , & Tiedemann, A. V. (2020). A global survey on diseases and pests in oilseed rape—Current challenges and innovative strategies of control. Frontiers in Agronomy, 2(October), 1–15. 10.3389/fagro.2020.590908

[gcbb12918-bib-0200] Zimmer, C. T. , Müller, A. , Heimbach, U. , & Nauen, R. (2014). Target‐site resistance to pyrethroid insecticides in German populations of the cabbage stem flea beetle, *Psylliodes chrysocephala* L. (Coleoptera: Chrysomelidae). Pesticide Biochemistry and Physiology, 108(1), 1–7. 10.1016/j.pestbp.2013.11.005 24485308

