# Supplementary material for: Integrated pest management strategies for cabbage stem flea beetle (Psylliodes chrysocephala) in oilseed rape
Source: Glob Change Biol Bioenergy. 2022 Jan 16;14(3):267–86. doi: 10.1111/gcbb.12918 (PMC9303719; doi:10.1111/gcbb.12918)
Supplement: Supplementary file 1 — Table S1 [file GCBB-14-267-s001.docx]

# Supplementary material

# Integrated pest management strategies for cabbage stem flea beetle (*Psylliodes chrysocephala*) in oilseed rape

Patricia A. Ortega-Ramos^1, 2^, Duncan J. Coston ^1,2^, Gaëtan Seimandi-Corda^1^ Alice L. Mauchline^2^ & Samantha M. Cook^1*^

*Corresponding author - sam.cook@rothamsted.ac.uk

^1^ Biointeractions & Crop Protection Department, Rothamsted Research, Harpenden, Hertfordshire, UK

^2^School of Agriculture, Policy and Development, University of Reading, UK

**Table S1 Control thresholds for adult and larval stages of cabbage stem flea beetle (*Psylliodes chrysocephala*) used in different countries on the European continent**

| **Country** | **Rapeseed production in 2019***  **(Tonnes)** | **Source** | **Threshold for control of adult feeding damage** | **Threshold for control of larval feeding damage** |
| --- | --- | --- | --- | --- |
| Albania | NI | CABI^a^ | Not present / no information available- | |
| Austria | 107,170 | BFL (2000) | 2-3 /m^2^ | - |
| Belgium | 33,430 | CABI^a^ | Not present / no information available | |
| Belarus | 578,068 | CABI^a^ | Not present / no information available | |
| Bulgaria | 432,490 | CABI^a^ | Not present / no information available | |
| Bosnia & Herzegovina | 8,657 | Bagi & Bondnar (2012)^b^ | 15 adults/day in yellow water trap or 0.5 adults /metre row or 2 adults per m^2^ | 2-3 larvae/ plant |
| Croatia | 103,900 | Maceljski (2002) | 15 adults/day in yellow water trap or > 2 adults/m^2^ | 2-3 larvae/plant until November if adults still active (temperatures between 4 -14 °C) |
| Czechia/  Czech Republic | 1156,970 | ÚKZÚZ supervisory authority of CZ Agriculture Ministry (2021)  Marek Seidenglanz (Pers. com.) | 1 adult per 1-metre row  10% leaf area eaten and / or 10 adults per yellow trap per 3-day period (or 3 adults/day). | 1 larvae/plant |
| Denmark | 729,000 | SEGS Crop & Environment | 10% leaf area eaten until 4-true leaf stage | 25 beetles caught in small yellow water traps (13 cm diameter) or 50 beetles caught in larger traps over 3-week period (based on 2 yellow water traps/10 ha) |
| Estonia | 191,370 | Eve Veromann (Pers. com.) | Bonnemaison & Jourdheuil (1954) state presence in Estonia, however records have not been confirmed in over 20 years (no pest status; no threshold) | |
| Finland | 41900 | Sari Himanen, Erja Huusela & Jarmo Ketola (Pers. com) | Species present on national database and known to occur in crops in southern areas but does not have pest status; no thresholds | |
| France | 3523300 | Robert, 2021 | 8/10 plants have shot holing symptoms and 25% of leaf area consumed before 4 leaf stage  If the crop was sown late (after October 1^st^) threshold reduced to 3/10 damaged plants | 2 - 3 larvae/plant |
| Germany | 2,830,200 | Godan, 1950;  Hoßfeld, 1987, 1993 | 10% leaf area eaten (Hoßfeld, 1987);  50 beetles per yellow water trap caught within three weeks during the main migration period (Hoßfeld, 1993) | 5 larvae per plant in autumn (Godan, 1950) |
| Greece | 9,680 | Petitpierre et al., (2017) | Species present but in low numbers (no pest status, no threshold) | |
| Hungary | 912120 | Bagi & Bondnar (2012) | 15 adults/day in YWT or 0.5 adults /metre row or 2 adults per m^2^ | 2-3 larvae/ plant |
| Iceland | NI | CABI^a^; Gia Aradottir (Pers. com) | Not present | |
| Ireland | 37,490 | TEAGASC; Michael Hennessy (Pers com.) | CSFB listed as a potential threat, guidance as per UK. | |
| Italy | 37,990 | Anon (2018) | 25-30% of plants with shot-hole symptoms up to 2-3 true leaf stage and /or 10% of leaf area eaten | 1 larva/plant |
| Kosovo | NI | CABI^a^ | Not present / no information available | |
| Latvia | 408,300 | Bukejs (2009) | Species present but very rare, recorded only in three localities (no pest status; no threshold) | |
| Lithuania | 692,480 | Bukejs et al., (2012 | First record of the species since 1936 so species present but very rare (no pest status, no threshold) | |
| Luxembourg | 10,060 | Luxembourg Institute of Science & Technology Bulletins^c^ | 10% leaf area eaten or  50 beetles per yellow water trap caught within three weeks during the main migration period | 5 larvae per plant in autumn |
| North Macedonia | 124 | Acronistrueimage (2020) | Pest present but thresholds available for vegetable brassicas only (no pest status; no threshold) | |
| Moldova | 77,268 | CABI^a^ | Not present / no information available | |
| Montenegro | NI | CABI^a^  Snježana Hrnčić (Pers. com.) | Not present / no information available  OSR not grown commercially on any significant scale; no advised thresholds | |
| Netherlands | 6,010 | CABI^a^  Weusthuis (2008) | Feeding damage to 10% of the leaf area eaten in autumn; at 4 to 5-leaf stage: 50 beetles/yellow waer trap in 3 weeks;  or 2 adults per m^2^ | 3-5 larvae/plant |
| Norway | NI | Annette Folkedal Schjøll (Pers. com.) | Present, but not considered a pest | |
| Poland | 2,268,850 | Poradnik (2018) | 3 adults per 1-metre row | 1 - 2 larvae per plant |
| Portugal | NI | Afonso & Cavaco (2009) | Species not listed as pest of oilseed rape (no pest status, no threshold) | |
| Romania | 798,220 |  | No information available | |
| Slovenia, | 9,450 | Kmetijsko gozdarska zbornica Slovenije (2000) | 50% plants have >2 shot holes /plant at cotyledon growth stage |  |
| Slovakia | 422,230 | ÚKZÚZ supervisory authority of CZ Agriculture Ministry^b^ | 1 adult per 1-metre row | 1 larvae/plant |
| Serbia | 84,311 | Bagi & Bondnar (2012)  Anon (2016) | 0.5 adults per 1-metre row or 2 adults per m^2^  >15 adults per yellow trap per day. | 2-3 larvae per plant during November |
| Spain | 144,770 | DEKALB (2016)  Pioneer (2017)  Lezaun et al., (2004) | 3/10 plants with feeding damage (before 3-true leaf growth stage) | 7/10 plants have larvae |
| Sweden | 381,500 | Jordbruksverkets växtskyddscentraler (Swedish board of Agriculture) (2021), | 5% leaf area loss at cotyledon stage; >10% leaf loss at 1-2 true leaf stage | 50-100 adults per yellow water trap, accumulated over three weeks  1-2 adults per 1-metre row, as determined by walking with a flashlight at night |
| Switzerland | 67,843 | Agridea (Swiss Centre for Agricultural Advisory and Extension Services)  Derron & Goy (1991) | At cotyledon stage, 50% of 50 sampled plants (n=50; 5 x 10 plants in different occasions) show shot-hole feeding damage; At 5-6 true leaves if 80% plants damaged | If >100 adults caught in yellow water trap within 3 weeks; or 70% (n=10) plants have >1 larva |
| UK | 1,752,000 | AHDB (2021)  Green, (2008). | During emergence: if risk is high, consider treatment at the first sign of attack  25% of leaf area damaged at the cotyledon - 2 leaf growth stage;  50% leaf area damaged at the 3–4 leaf stage  or when the crop is growing more slowly than it is being eaten | 5 larvae/plant  50% leaf petioles damaged  Average of >100 beetles per yellow water trap in weekly monitoring from September until October |
| Ukraine | 3,280,320 | Pokoziy et al (2010) | Not listed as a pest of oilseed rape (no pest status, no threshold) | |

*OSR production data (2019) obtained from: FAOstats <http://www.fao.org/faostat/en/#data/QC>

NI - No data available

^a^Presence data from CABI Invasive Species Compendium Distribution Maps (Psylliodes chrysocephala). https://www.cabi.org/ISC/datasheet/116582.

^b^Information published in Serbia, in collaboration with Hungary, but the pedo-climatic conditions where oilseed rape is grown in Bosnia are almost identical and local advisors use the same recommendations (Dimitrije Markovic, Pers. com.).

^c^Pedoclimatic conditions similar to Germany, so those thresholds adopted (Michaël Eickermann, Pers. com.)

^d^The majority of thresholds for common pests are usually adopted by Slovak authorities from Czech recommendations (historically, they were one joint authority) (Daniel Nerad, Pers. com.)

**References**

# Acronistrueimage (2020). како да се ослободите од градиното месо. како да се справи со раселеното месо на зелката и на издистењето. знаци на инвазијата на болвата на зелката 13.04.2020. <https://acronistrueimage.ru/mk/huawei/kak-izbavitsya-ot-sadovoi-bloshki-kak-pravilno-borotsya-s-krestocvetnoi/>

Afonso, M. J., & Cavaco, M. (2009). Manual de protecção fitossanitáriaem protecção integrada e agricultura biológica de culturas oleaginosas. <https://www.dgav.pt/wp-content/uploads/2021/01/MANUAL-DE-PROT-FITOSS-EM-PROT-INTEGR-E-AGRIC-BIOLOG-DE-CULT-OLEAGINOSAS.pdf>

AHDB (2021) Cabbage stem flea beetle (CSFB) treatment thresholds in oilseed rape. 21June 2021. <https://ahdb.org.uk/knowledge-library/cabbage-stem-flea-beetle-csfb-treatment-thresholds-in-oilseed-rape>

Agridea (2021). Bekämpfungsschwellen für Massnahmen gegen die Schadorganismen im Feldbau (im ÖLN obligatorisch). https://www.agridea.ch/fileadmin/AGRIDEA/Theme/Productions_vegetales/Grandes_cultures/bekaempfungsschwellen/1.0.3-1.0.9_Bekaempfungsschwellen_2019.pdf

Anon (2016). Poljoprivrednik, Issue 2597, September 2016, Novi Sad, Serbia. <https://www.poljoprivrednik.net/nasa-izdanja/poljoprivrednik/2025-naslovi-iz-broja-2597>

Anon (2018). ‘Norme tecniche applocative’ Sistema di Qualita’ Nazionale Produzione Integrata - disciplinari di produzione integrata difesa integrata. Regione Molise Dipatimento Governo del Territorio, Mobilita’ e Risorse Naturali, 140

Bagi, F. & Bondnar, K. (2012). Fitomedicina. Pg 148-50. Publisher: University of Novi Sad, Novi Sad, Serbia. ISBN: 978-86-7520-234-9

BFL (Bundesamt und Forschungszentrum für Landwirtschaft (2000). Richlinien für die pflanzenschutzarbeit. BMLF, Vienna.

Bonnemaison, L., & Jourdheuil, P. (1954). L’altise d’hiver du colza (*Psylliodes chrysocephala* L.). Ann Épiphyties, 4, 345–524.

Bukejs, A. (2009). To the knowledge of flea beetles (Coleoptera: Chrysomelidae: Alticinae) of the Latvian fauna. 5. Genus Psylliodes Latreille, 1825.Latvijas entomologs 47: 6-15.

Bukejs, A., Tamutis ,V., Ferenca, R., & Alekseev, V.I. (2012). New and insufficiently known leaf-beetle species (Coleoptera: Chrysomelidae) of the Lithuanian fauna. 2. Zoology and Ecology. 22(3-4):203-11.

# CABI, Invasive Species Compendium (2021). *Psylliodes chrysocephala* (cabbage stem flea beetle). <https://www.cabi.org/ISC/datasheet/116582>

DEKALB Spain (2021). Boletín informativo colza. ﻿https://www.dekalb.es/documents/89430/300232/La+lucha+contra+los+limacos+y+el+control+de+las+pulguillas+en+colza/a8dbad72-9380-4f8c-8ffd-ec1c6a4ca9b8

Derron J.O., & Goy G. (1991). L’altise d’hiver du colza (Psylliodes chrysocephala L.): biologie, nuisibilité et moyens de lutte. Revue Suisse d’Agriculture, 23, 5-9

Godan, D. (1950). Wann ist der Rapserdflohlarven-Befall für den Rapsacker gefährlicher, im Herbst oder im Frühjahr? Nachrichtenblatt des Deutschen Pflanzenschutzdienstes (2), 149.153.

Green, D. B. (2008). Revised Thresholds for Cabbage Stem Flea Beetle on Oilseed Rape, HGCA Project report 428.HGCA, UK.

Hoßfeld, R. (1987). Schadschwellen bei tierischen Rapsschädlingen 5 (2), 70–72.

Hoßfeld, R. (1993). Die Gelbschale als Entscheidungshilfe bei der Bekämpfung des Rapserdflohs (*Psylliodes chrysocephala* L.). Gesunde Pflanzen 45 (8), 291–295.

Jordbruksverkets (2021). Bekämpningsrekommendationer Svampar och insekter 2021 page 46: [https://www2.jordbruksverket.se/download/18.51bb92a1179a14bd85280fe9/1622101854696/be17v30.pdf](https://eur01.safelinks.protection.outlook.com/?url=https%3A%2F%2Fwww2.jordbruksverket.se%2Fdownload%2F18.51bb92a1179a14bd85280fe9%2F1622101854696%2Fbe17v30.pdf&data=04%7C01%7Csam.cook%40rothamsted.ac.uk%7Cdf4bc9f15f644e2c7bba08d95bcea8f3%7Cb688362589414342b0e37b8cc8392f64%7C1%7C0%7C637641765423519433%7CUnknown%7CTWFpbGZsb3d8eyJWIjoiMC4wLjAwMDAiLCJQIjoiV2luMzIiLCJBTiI6Ik1haWwiLCJXVCI6Mn0%3D%7C1000&sdata=WZo4F7H04prI66%2Fgu3Fj3KwGW2DHO10NBO9jd7jnLOU%3D&reserved=0)

Kmetijsko gozdarska zbornica Slovenije (2000). Varstvo oljene ogreščice v pridelovalni sezoni 2020/2021. Publisher Kmetijsko Gozdarski Zavod Celje, Oddelek za kmetijsko savetovanje, Celje, Slovenia. [https://www.kmetijskizavod-celje.si/uploads/kgzs_-_zavod_ce/varstvo_rastlin/varstvo_oljne_ogrscice_jesen_2020.pdf](https://eur01.safelinks.protection.outlook.com/?url=https%3A%2F%2Fwww.kmetijskizavod-celje.si%2Fuploads%2Fkgzs_-_zavod_ce%2Fvarstvo_rastlin%2Fvarstvo_oljne_ogrscice_jesen_2020.pdf&data=04%7C01%7Csam.cook%40rothamsted.ac.uk%7C2900cbddbcbd434ac80e08d8eace1c60%7Cb688362589414342b0e37b8cc8392f64%7C1%7C0%7C637517518248827855%7CUnknown%7CTWFpbGZsb3d8eyJWIjoiMC4wLjAwMDAiLCJQIjoiV2luMzIiLCJBTiI6Ik1haWwiLCJXVCI6Mn0%3D%7C1000&sdata=%2BXVI9%2FXMMqQ0ga9qNZ1qtw4dxfZ4urTtHmYHm3o6UGY%3D&reserved=0)

Lezaun, J.A., Armesto, A.P., & Lafarga, A. (2004). Problemática y sistemas De protección del cultivo malas hierbas. Navarra Agrar 28–34.

Maceljski, M. (2002). Poljoprivredna entomologija. Zrinski, Čakovec.

Petitpierre, E., Sacares, A., & Jurado-Rivera J.A. (2017). Updated checklist of Balearic leaf beetles (Coleoptera: Chrysomelidae). Zootaxa. 4272(2):151-77.

Pioneer Spain (2015). Forum Pioneer Colza. Viewed 6 June 2021, https://www.pioneer.com/CMRoot/International/Spain/images/News/folleto_forum_colza_2015.pdf

Pokoziy Y.T., Pisarenko V.M., Dovgan S.V. et al (2010) Кyiv: Agrarna osvita, /Monitoring of pests of agricultural crops: a textbook / 223 p. ISBN 978-966-7906-94-8

Poradnik Sygnalizatora Ochrony Rzepaku. (2018). Tratwal A., Strażyński P., Jajor E., Mrówczyński M. Wydawnictwo Instytut Ochrony Roślin - PIB, Poznań: 78 - 81.

Robert, C. (2021). Gestion en cours de campagne des grosses altises adultes (altises d’hiver). Terres Inovia. ﻿<https://www.terresinovia.fr/-/surveillance-et-lutte-contre-la-grosse-altise>

SEGES (Landbrug & Fødevarer) <https://en.seges.dk/>

TEAGASC Cropquest Oilseed rape: Crop Report: <https://www.teagasc.ie/crops/crops/research/programme-activities/cropquest/oil-seed-rape-osr/>

ÚKZÚZ supervisory authority of CZ Agriculture Ministry (2021) UKZUZ Crop plant pest catalogue/portal:

<https://eagri.cz/public/app/srs_pub/fytoportal/public/#rlp%7Cso%7Cskudci%7Cdetail:f50546d2ac767ccc6ca48bbc1a20efac>

Weusthuis, M. (2008) Teelthandleiding koolzaad. Weusthuis Agri Servics, 41pg.

**Acknowledgements**

Sincere thanks to the following for supplying information on thresholds for CSFB control: Dimitrije Markovic & Danijela Kondic (Bosnia & Herzegovina) (& Macedonia, Montenegro); Ivan Juran (Croatia); Daniel Nerad & Marek Seidenglanz (Czech Republic) (& Hungary, Slovakia) ; Emily Bick & Helle Mathiasen (Denmark); Eve Veromann (Estonia) (& Latvia, Lithuania); Sari Himanen, Erja Huusela & Jarmo Ketola (Finland); Daniel Rüde & Bernd Ulber (Germany); József Vuts (Hungary); Michael Hennessy (Ireland); Francesco Pennacchio (Italy); Femke de Jong & Femke Van Den Berg (The Netherlands); Stanislava Lazarevska (North Macedonia); Snježana Hrnčić (Montenegro); Ishita Ahuja & Annette Folkedal Schjøll (Norway); Małgorzata Jędryczka (Poland); José Paulo Sousa (Portugal); Milan Plećaš (Serbia) (& Slovenia, Macedonia); Stanislav Trdan (Slovenia); Ola Lundin (Sweden); Giselher Grabenweger (Switzerland); Bykov Mykola & Roman Grynyshyn (Ukraine).
